# Genetic diversity and population structure of native maize populations in Latin America and the Caribbean

**DOI:** 10.1371/journal.pone.0173488

**Published:** 2017-04-12

**Authors:** Claudia A. Bedoya, Susanne Dreisigacker, Sarah Hearne, Jorge Franco, Celine Mir, Boddupalli M. Prasanna, Suketoshi Taba, Alain Charcosset, Marilyn L. Warburton

**Affiliations:** 1 International Maize and Wheat Improvement Center, Applied Biotechnology Center, Texcoco, Mexico; 2 Universitat de les Illes Balears, Departament de Biologia, Illes Balears, Spain; 3 Universidad de la Republica, Facultad de Agronomía, Estadística y Computación, Paysandú, Uruguay; 4 Unité Mixte de Recherche de Génétique Végétale, Institut National de la Recherche Agronomique–Université Paris Sud–Centre National de la Recherche Scientifique -AgroParisTech, Yvette, France; 5 United States Department of Agriculture, Corn Host Plant Research Resistance Unit, Mississippi State University, Mississippi State, MS, United States of America; National Cheng Kung University, TAIWAN

## Abstract

This study describes the genetic diversity and population structure of 194 native maize populations from 23 countries of Latin America and the Caribbean. The germplasm, representing 131 distinct landraces, was genetically characterized as population bulks using 28 SSR markers. Three main groups of maize germplasm were identified. The first, the Mexico and Southern Andes group, highlights the Pre-Columbian and modern exchange of germplasm between North and South America. The second group, Mesoamerica lowland, supports the hypothesis that two separate human migration events could have contributed to Caribbean maize germplasm. The third, the Andean group, displayed early introduction of maize into the Andes, with little mixing since then, other than a regional interchange zone active in the past. Events and activities in the pre- and post-Columbian Americas including the development and expansion of pre-Columbian cultures and the arrival of Europeans to the Americas are discussed in relation to the history of maize migration from its point of domestication in Mesoamerica to South America and the Caribbean through sea and land routes.

## Introduction

Maize was domesticated about 9000 years ago in Mexico from tropical teosinte, *Zea mays* ssp. *parviglumis*, in the Balsas River region in western Mexico [1,2]. The ultimate expression of maize domestication and subsequent diffusion was its diversification into numerous varieties, called landraces, each of which has acquired distinct genetic and morphological characteristics mainly due to local adaptation and human selection [3]. The dispersion of maize throughout Mesoamerica, region of ancient civilizations and native cultures before the arrival of the Spanish, to North and South America and the Caribbean followed different routes, probably related to the migration of archaic peoples and later linked to the complex systems of exchange between cultures developed in pre-Columbian America.

**Fig 1:**
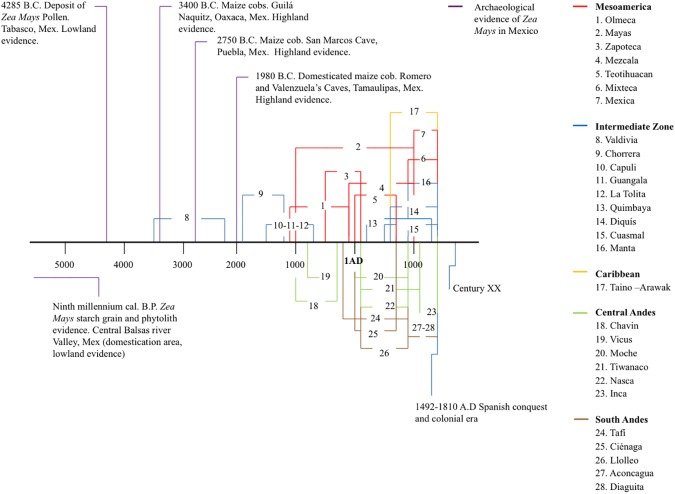
Historical timeline of pre-Columbian maize cultivation by geographical region and some Pre-Columbian cultures linked with maize cultivation. Figure created using available historical and archeological records [4,5,6,7,8].

Many archaeological sites in the Americas have evidence of sedentary communities linked to maize cultivation, some in Mesoamerica as early as nearly 9000 years ago (Fig 1). The Americas were home to multiple pre-Columbian cultures, defined according to geographical, ethnic and linguistic characteristics. In the Mesoamerican region, Olmec culture developed along the Gulf of Mexico (1200–500 BC.). The Maya (1100 BC-1000 AD) and Zapotec cultures (500 BC-900 AD) arose in Southern Mexico and Guatemala, a large geographic region with great environmental variability. In the highlands of Central Mexico, the Teotihuacan (1–600 AD), Toltec (800–1100 AD), and Mexica (1200–1520 AD) cultures emerged.

An intermediate cultural region existed between Mesoamerica and northern South America, in which the Valdivia and Chorrera cultures (among others) were established. Both these cultures are associated with early cultivation of maize (3500–300 AD), geographically far from maize’s domestication origin. In South America, several cultural regions can be identified, including the Amazonian and Chaco regions. The central Andes were the site of the emergence of the Chavin, Vicus, Moche, Tiwanaco, Nazca, and Inca cultures (1000–1532 AD). The southern Andes encompass southern Bolivia, northwest Argentina, and the southern section of the Andes along Chile; cultures including the Aconcagua, Diaguita, and Tafi (300–1532 AD) can be linked to this region. The Caribbean and northern Venezuela are principally linked with the Taino-Arawak culture (450–1500 AD) [4,8].

Investigation of the spread of maize from its center of domestication has been extensive, based on cytogenetic data [9], historical and anthropological-ethnographic studies [10,11], archeological studies [4,6,7,12,13], and genetic data [1,3,14,15,16,17]. A SNP dataset was scored in a large number of accessions of both teosinte and maize to highlight new geographic elements of the earliest cultivated maize in America [16]. Finally, more recently, a multidisciplinary approach was used in order to reconstruct possible global patterns of maize diffusion out of the Americas [17].

Archeological studies include the study and dating of macrobotanical remains, phytoliths (the small opal silica bodies found in the cells of most of plants), and pollen samples recovered from sediments in lakes, wetlands, and archaeological deposits [4,6,7]. Key developments in two different branches of science, genetics and archaeology, have shed light on the early domestication and dispersal of maize, including the accumulation of genetic evidence that maize was domesticated from an annual Balsas teosinte [1]; and the ability to date maize fragments and phytoliths. These developments have allowed the formulation and documentation of the early history of maize including location and chronology for domestication and early dispersal [4,6]. Documentation exists that maize was being grown during the early ninth millenium B.P. in the Balsas River Valley [6]; however, information on early maize history in the archaeological record is incomplete, and in particular there are discrepancies regarding the earliest presence of maize in the Andes, partly related to whether dates were inferred or directly calculated [18]. Some studies suggest an early (7000 B.P.) introduction of maize into the western part of South America [7,12], but another suggests a later introduction, between 4000 and 3500 B.P. [19].

Questions remain regarding the domestication event itself. Evidence (and obvious final outcome) of the intentional human selection for increased cob and grain size in maize [20] indicates that the major focus of maize utilization was directed toward the cob of the plant [6]. However, it is also possible that early domestication was based on the nutritional value of stems and seeds as a source of sugar that could be extracted by chewing, or for the production of alcoholic beverages [21]. Regardless of the original intended use, the transition from the economy of hunter-gatherers to that of food producers, and from nomadic to more sedentary lifestyles, including increasingly larger population centers, is linked to the emergence of agriculture based on maize (and squash, beans, and chilies) in Central America [22].

Considering that Mexico is the center of origin and one main center of diversity of maize, and further considering the cultural importance of maize, genetic variability in Mexico has been thoroughly studied since 1913 [23,24]. The concept of the racial complex for the classification of Mexican maize landraces was established in the early 1950s [25]. Later, using a comprehensive review of previous research [26], Mexican landraces were placed into three main groups: the first containing long and narrow ears that are found in northwestern Mexico and southwestern United States; another comprised of high elevation maize with conical ears; and the last group possessing maize with small and long ears typical from the lowlands from southern Mexico. This classification is accepted as correct by most researchers, but refinements and modifications have been suggested [23,24] (S1 Fig). Compared to the wealth of data available for Mexican landraces, landraces from the rest of Latin America have been very poorly characterized overall. This can be remedied with the use of molecular markers, including Simple Sequence Repeats, (SSRs), which have been used to characterize maize landraces in many diversity studies due to their multiple alleles per locus, ease of use, and good mutation signature for the time scale under study [1,3,14,27,28,29,30]. Due to the heterogeneous nature of maize landraces, genetic diversity analyses should involve a large number of individuals per landrace. Several genetic diversity studies have used the bulked method with SSRs, using one or two bulks of 8–15 individuals to more efficiently represent each population [15,31,32,33].

Understanding evolutionary history, genetic diversity within and among indigenous races, and relationships between the many traditional Latin American races of maize are all critical for fundamental research, conservation, and utilization of these genetic resources for maize breeding. Here, we hypothesize that morphological and genetic variation present in modern maize populations were influenced by geographical factors and reflect the distribution of human historical and cultural events in the New World. To this end, we performed genetic characterization using SSR markers of native Latin American maize populations, in order to study diversity and population structure and argue that they relate to the migration of maize from its center of origin in Mesoamerica towards South America and the Caribbean. We first analyzed SSR data from Mexican maize landraces alone, to validate the genetic data in germplasm for which very good historical and anthropological evidence are available. This was followed by an analysis of 194 maize landraces from all over Latin America, to clarify some of the gaps and disputes concerning early maize diffusion.

## Materials and methods

**Fig 2:**
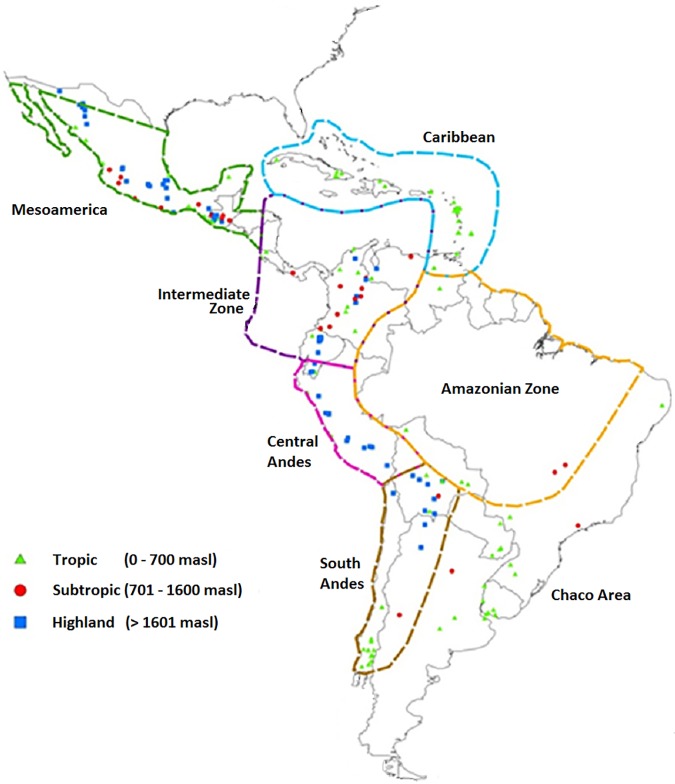
Geographic origins and typical altitude of growing environment of 194 maize native populations and extent of Pre-Columbian cultural regions in the Americas.

For this study, 194 native Latin American maize populations were selected from the CIMMYT Maize Germplasm Bank based on eco-geographical data to represent 131 classified landraces from 23 countries (Fig 2, S1 Table). A subset of this data set was included in a previous study, which evaluated these accessions with fewer markers but many more entries covering the global range of maize cultivation [17]. The year of collection for these accessions ranges from 1946 to 2000. In order to include the racial and geographical Mexican complexes defined in previous research [25,26], 27 landraces from the three major centers of traditional maize production were included (the Central Valleys, Sierra Madre Occidental, and Southern and Southwestern Mexico).

A population-level genotyping strategy was chosen to characterize the range of allelic diversity among and within populations [31]. Thirty seeds of each population were planted in the greenhouse and 10cm leaf fragments were harvested from 15 individual plants and bulked to form a composite sample representing each population. DNA was extracted from freeze-dried bulked leaves according to CIMMYT protocols [34]. DNA was quantified using absorbance at 260nm measured by a NanoDrop spectrophotometer (Thermo Scientific, Wilmington DE).

### Genotypic characterization

For genetic analysis, 28 SSRs that had been optimized previously to work in pooled samples of 15 individuals were selected (S2 Table). These SSRs gave good coverage of the maize genome with all linkage groups represented. Fragments (alleles) of each SSR were generated via PCR according to CIMMYT standard protocols [34]. Electrophoresis was conducted using an automatic capillary sequencer ABI 3100 (Applied Biosystems, Foster City, CA) to separate and size the fragments. Data were analyzed using the programs GeneScan ® 3.1 (PerkinElmer / Applied Biosystems, Foster City, CA) and Genotyper ® 2.1 (PerkinElmer / Applied Biosystems, Foster City, CA) to generate a data set of all fragments including size in base pairs, peak height (corresponding to intensity of the amplified fragment), and quality score.

### Data analysis

Frequency of each allele in the bulk was calculated from peak height (intensity) in *R*, an open-source computer program and language for data analysis (http://cran.r-project.org), using the program Freqs-R [35]. This program removes false peaks caused by PCR stuttering or preferential amplification. For each pooled sample, the *R* program FtoL-R [36] was used to simulate the alleles (calculated as length in base pairs) of 15 individuals to meet allele frequencies and expected heterozygosity of each sample, for analyses requiring genotypic data for individuals, rather than population allelic frequencies. Number of alleles, genetic diversity, and genetic distance between populations (from proportion of shared alleles) were calculated using the program PowerMarker [37], for each accession or defined subgroup. The program Darwin 5.0 [38] was used for cluster analysis using the Neighbor-Joining method, and Principal Coordinate Analyses (PCoA) based on the genetic distances matrix obtained from PowerMarker.

The model-based clustering method, Structure 2.2 [39] was used to analyze population structure and identify sub-groups within the overall set of populations. Assumptions were set to an admixture model in which *K* populations/groups were characterized by a set of allele frequencies at each locus. Populations were not assigned to any group *a priori*, and individual simulations of each population were allowed to vary. After the first analysis including all populations, additional Structure analyses were performed in order to reveal possible sub-structure within each of the main clusters detected. Linked to the Structure software package an additional calculation was used to determine the most suitable number of clusters or subpopulations taking into account the values obtained for*ΔK* (supplemental methods).

Preliminary classification of the Mexican landraces was compared to reported classification studies available for these landraces (S3 Table), based on the three main racial complexes [26] as the best standard. For the 38 Mexican populations, probabilities for *K* were calculated from 1 to 8, and for the entire data set, including all populations, from 1 to 15. Calculations were performed using 1,000,000 replications after a burn in period of 500,000 iterations, and the procedure was repeated five times for each *K* value. Populations were then assigned to each group for which they had an ancestry proportion *Q*_*jk*_ greater than 51.0%; if a population did not show an ancestry proportion higher than this value, it was assigned to the mixed group.

## Results

### SSR classification validation in Mexican maize germplasm

**Fig 3:**
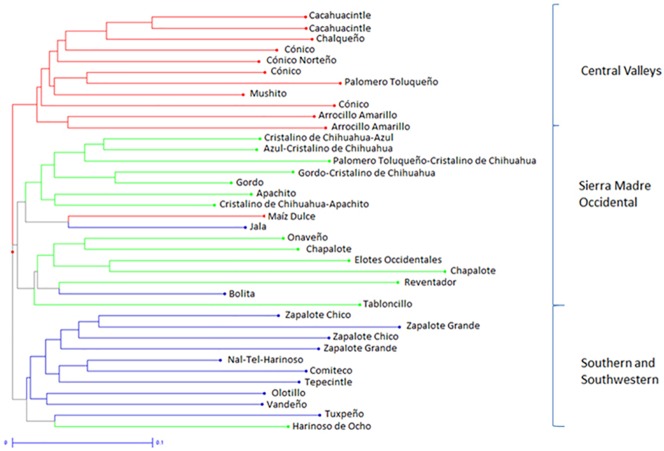
Neighbor-joining cluster analysis of 38 maize landrace populations from Mexico based on proportion of shared alleles from 28 SSR markers. Populations are labeled according to race name and are colored according to the classification of racial complexes [26].

The relationships between the Mexican maize races can be seen in the cluster analysis in Fig 3. The first group (Sierra Madre Occidental (SMO), in green) is typical of the Sierra Madre Mountains in northwestern Mexico. The second group (Southern Mexico (S)), in blue represents the large eared maize from southern and southwestern Mexico. The third group (Central Valleys (CV)), in red includes the landraces with conical ears found in the highlands of central Mexico. Good separation and very little overlap are seen among these three sub-populations, which agree very well with previous classifications (S3 Table), as well as with the PCoA (data not shown). Four landraces (Jala, Bolita, Harinoso de Ocho and Maiz Dulce) do not fall in the same groups reported for the racial complexes [26]. This may be due to specific traits and pedigrees characteristic of these particular populations. The putative parents of Bolita and Jala come from different racial complexes found here [25, 28], and Jala has been traditionally known for its uniqueness, producing the longest maize ears in the world. Harinoso de Ocho is considered an ancient landrace that has had wide influence on populations ranging from northwestern to southeastern landraces [9, 25]. Maiz Dulce has special status due to its inconsistent grouping in previous studies and sometimes is considered as a separate race [28]. Strong selection, and possible genetic drift, may have changed these landraces over time. It may be likely that classification based on particular characteristics strongly influenced by human selection may not accurately represent genetic relationships between defined races. In addition, previous classifications based on only one or a few individuals may have missed allelic diversity that is likely to be captured in a bulked analysis of more individuals, such as was used in this study.

Four clusters in Structure were assumed based on *ΔK* (S2 Fig), and the results obtained were highly consistent with the NJ cluster analysis and the PCoA graphical representation of Mexican landrace populations. Specific groups were identified based on geography and racial complexes (Table 1). At *K* = 4, 95% of the populations were assigned to groups, which highlighted the three racial complexes (CV, S, and SMO) found in cluster analysis, plus one additional group, a Chapalote related complex (r-CH, excluding one landrace, 142, which clustered with the SMO). Similarly, the races of western and northwestern Mexico were found to belong to a related group, and the populations in the Chapalote complex (including Chapalote and Reventador and the Elotes Occidentales) were highly interrelated [40]. In agreement with the results of the dendrogram in Fig 3, populations 149 and 161 from the races Bolita and Maiz Dulce did not cluster as expected based on previously assigned race (Table 1). However, when *K* was set to six, the structure was more specific and corresponded better with phenotype.

**Table 1:** Assignment of the Mexican populations in each group determined by population structure analysis for *K* = 4 and *K* = 6.

ID	Race name	*K* = 4	*K* = 6
125	Gordo	SMO	ESMO
126	Azul > Cristalino de Chihuahua	SMO	ESMO
127	Palomero Toluqueño > Cristalino de Chihuahua	SMO	ESMO
128	Cristalino de Chihuahua>Apachito	SMO	ESMO
129	Cristalino de Chihuahua>Azul	SMO	ESMO
130	Apachito	SMO	ESMO
131	Zapalote Grande	S	ZC
132	Comiteco	S	S
133	Palomero Toluqueño	CV	CV
134	Cacahuacintle	CV	CV
135	Chalqueño	CV	CV
136	Mushito	CV	CV
137	Harinoso de Ocho	SMO	WSMO
138	Zapalote Chico	S	ZC
139	Zapalote Chico	S	ZC
140	Cónico	CV	CV
141	Arrocillo Amarillo	CV	CV
142	Chapalote	SMO	WSMO
143	Onaveño	SMO	WSMO
144	Conico	CV	CV
145	Arrocillo Amarillo	CV	CV
146	Nal-Tel>Harinoso	S	S
147	Zapalote Chico	S	ZC
148	Chapalote	r-CH	r-CH
149	Bolita	30.7(S) 37.8(SMO) 16.0(r-CH) 15.5 (CV) ^§^	WSMO
150	Conico	CV	CV
151	Tuxpeño	S	S
152	Tepecintle	S	S
153	Vandeno	S	S
154	Elotes Occidentales	r-CH	r-CH
155	Jala	SMO	ESMO
156	Reventador	r-CH	WSMO
157	Olotillo	SMO	S
158	Gordo>Cristalino de Chihuahua	SMO	ESMO
159	Cónico Norteño	CV	CV
160	Tabloncillo	SMO	ESMO
161	Maíz Dulce	12.3(S) 26.0(SMO) 50.6(r-CH) 11.0(CV) ^§^	ESMO
162	Cacahuacintle	CV	CV

^***§***^ Percentage of population’s ancestry that does not belong to any group, taking an arbitrary ancestry cutoff of 51%.

Abbreviations: Central Valleys (CV), Sierra Madre Occidental (SMO), Southern Mexico (S), Chapalote related complex (r-CH), eastern slope of Sierra Madre Occidental (ESMO), western slope of Sierra Madre Occidental (WSMO) and the Zapalote Complex (ZC).

In general, the same clusters were seen, except that the Sierra Madre Occidental cluster divided into populations from the eastern slope (ESMO) and western slope (WSMO). The ESMO landraces are limited to the northern and westernhighlands of Mexico (mainly in Chihuahua and some parts of Sonora, Durango and Jalisco) in small valleys from altitudes from 2000 to 2600 masl. The WSMO landraces are comprised of mostly eight-row landraces (those with eight rows of kernels, as opposed to the average 16 rows) distributed in low elevations in the west and northwest of Mexico [23]. The Southern Mexico (S) cluster contains landraces grown at medium to low altitudes, including Tropical Dents (the agronomically important progenitors of many modern maize varieties, Tuxpeño, Vandeño, and Tepecintle), Tropical Early (Nal-Tel), and Tropical Late (Olotillo, Comiteco). This cluster contains the Zapalote racial complex as well, including populations of Zapalote Chico and Zapalote Grande. The next cluster included the Chapalote related complex (r-CH), comprised of landraces grown at elevations of 100 to 500 masl in the Pacific Coastal Plain of Nayarit to Sonora [23]. Finally, the Central Valleys (CV) cluster agreed with the dendrograms, still containing the landraces from the highlands of central Mexico. The classification of Mexican landraces based on the bulked analysis of SSRs used in this study was in good agreement with previous classifications made using molecular markers and morphological characteristics.

### Classification of Latin American maize germplasm

**Fig 4:**
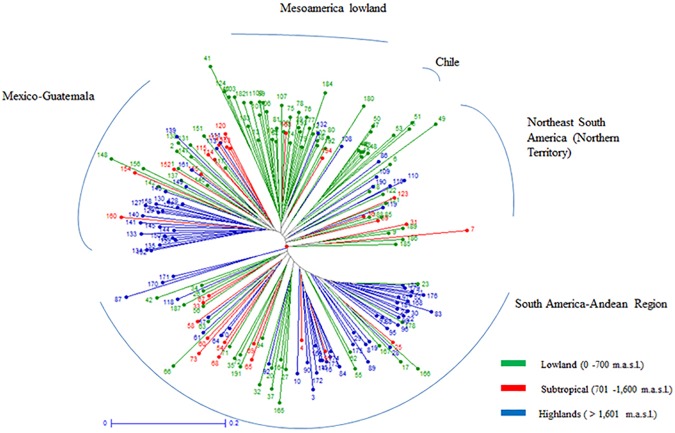
Neighbor-joining cluster analysis of 194 maize populations from Latin America and the Caribbean based on proportion of shared alleles from 28 SSR markers. Populations are labeled according to ID number and colored according to altitude where the accessions were grown.

**Fig 5:**
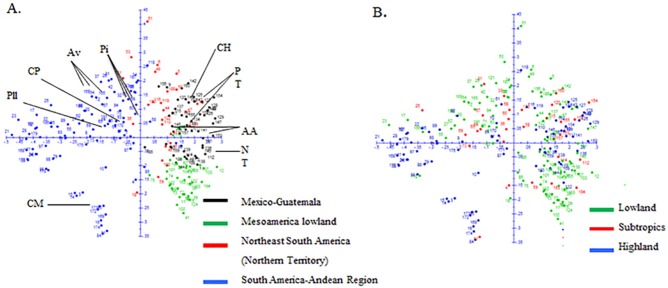
Relationships of 194 native populations from Latin America and the Caribbean revealed by Principal Coordinate Analysis based on proportion of shared alleles. (A) populations identified with ID number and colored according to groups found in cluster analysis (Fig 4), and the ancient indigenous landraces are indicated: Palomero Toluqueño (PT), Arrocillo Amarillo (AA), Chaplaote (CH) and Nal-Tel (NT) from Mexico, Pollo (Pll) and Pira (Pi) from Colombia, Avati (Av) from Paraguay, and Confite Morocho (CM), and Confite Puneño (CP) from Peru. (B) populations identified with ID number but colored according to altitude where the accession was collected.

Cluster analysis of all populations in the study identified four groups loosely based on geography (Fig 4), including a group of Mexican and Guatemalan populations; a second group of southern Mexico, Central America and Caribbean populations (Mesoamerica lowland); a cluster of populations from eastern South America; and a cluster of Andean populations. The PCoA in Fig 5, while in good agreement with the cluster analysis, appeared to separate populations based on growing environment (altitude) as well as geography, suggesting adaptive influences.

The Eastern South American group found in the cluster analysis contained some populations adapted to a range of altitudes from Central America, and is similar in constitution to the group named "Northern Territory" in a previous classification [9]. In the present study, this cluster includes one Canguil race from Ecuador, Chilean maize populations, and the Bolivian Perola race. These populations are related to others from the eastern coast of South America including the Catetos from Brazil, Argentina and Uruguay, which are morphologically similar to the Coastal Tropical Flint from the Caribbean [41]. These relationships can also be seen in the PCoA analysis (Fig 5) between Mesoamerica lowland and the eastern South American cluster.

The cluster containing the 79 South American populations (Blue entries, Fig 4), was composed of all the Columbian populations except for one from race Amagaceño, all populations from Peru, Paraguay, and Ecuador (excluding Canguil mentioned above), the Bolivian populations (excluding Perola), populations from Brazil, Uruguay, Chile, and Argentina, one of four Venezuelan populations of the Cariaco race, and, unexpectedly, one population from Guatemala. Although some Columbian races are morphologically similar to Mexican races and have been considered pre-Columbian introductions [25] in this analysis, they are not directly related to the current Mexico or Central American races. The PCoA shows that the ancient indigenous landraces from Mexico including Palomero Toluqueño, Arrocillo Amarillo, Chapalote and Nal-Tel are not directly associated with other South American races considered as primitive, such as races Confite Morocho, Kully, and Confite Puneño from Peru [42], Pollo and Pira from Colombia [43] or Avati' Moroti and Avati' Pichinga from Paraguay [44]. This evidence further supports a temporally long separation between the ancient races of Mexico and South America.

**Fig 6:**
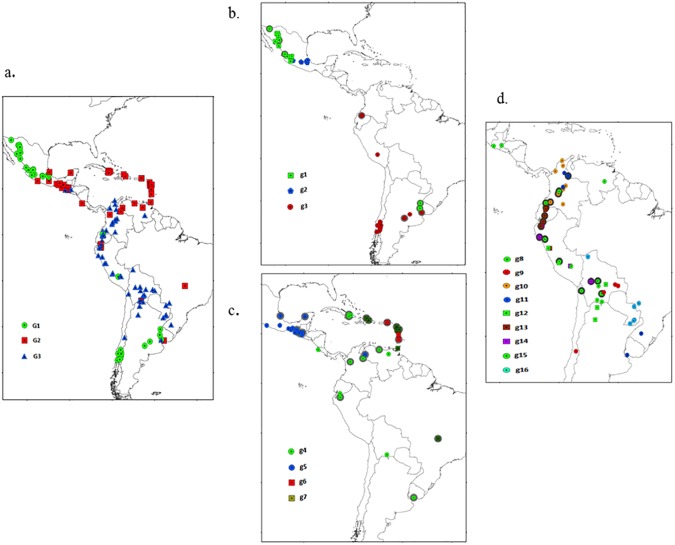
Geographical distribution of 194 maize populations from Latin America and the Caribbean estimated by population structure analysis. (a) Structure analysis for all 194 accessions that identified three main groups: Mexico and Southern Andes group (G1), Mesoamerica lowland (G2), and South America-Andean region (G3). (b) sub-structure analysis of G1 that identified three sub-clusters: northern Mexico (g1), central Mexico (g2) southern Andes (g3). (c) sub-structure analysis of G2 that identified 4 sub-groups: tropical lowland (g4), South Mex/Guat/Vir IS/Ven (g5), Lesser Antilles (g6), Greater and Lesser Antilles (g7). (d) sub-structure analysis for G3 that identified nine sub-clusters: Bolivian highlands (g8), Bolivian lowlands (g9), Columbian 1 (g10), Columbian 2 (g11), Highland Andean (g12), Ecuadorian highlands (g12), Central highlands Andean (g13), northern tropical lowlands (g15) and Moroti’ sub-cluster (g16). Proportion of ancestry cutoff offset to *Q*>51% for structure and sub-structure analyses. The less representative accessions in the sub-structure analyses accessions are labeled with gray shadow (51% < Q < 80%).

A Structure analysis run on all 194 populations at *K* = 3 assigned 87% of the populations to one cluster (S3 Fig). Independent Structure analyses within each of these three main clusters allowed the detection of different sub-clusters (Fig 6, S4–S6 Figs). The first Structure cluster (G1) was partitioned into three sub-clusters: northern Mexico (g1), central Mexico (g2), and southern Andes (g3) including races from the southern lowlands of Chile and Argentina, the Canguil race from Ecuador, Confite Puneño and San Jeronimo from Peru, and Cateto Sulino from Uruguay. The second main cluster, Mesoamerica lowland (G2), consisted of populations from southern Mexico, Guatemala, Costa Rica and Panama, all the Caribbean accessions, populations from northern Venezuela, the Tusilla race from Ecuador and the Cateto Nortista race from Brazil. These separated into four sub-clusters, (g4—tropical from Central America to Uruguay; g5—tropical races from the Caribbean, Venezuela, southern Mexico and Guatemala; g6—early introductions into the Caribbean; and g7—races from the Caribbean and Venezuela and some accessions that did not group at the sub-cluster level (Table 2).

**Table 2:** Sub-cluster composition based on sub-structure analysis for Mexico and Southern Andes (G1), Mesoamerica Lowland (G2) and the South America-Andean region (G3). Arbitrary ancestor cutoff of 51% according to Structure.

**Sub-cluster name**	**G1- Mexico and southern Andes**
**Northern Mexico (g1)**	Camelia(A)*; Gordo*,*Palomero Toluqueño > Cristalino de Chihuahua*, *Azul(2) > Cristalino de Chihuahua*,*Cristalino de Chihuahua>Apachito(2)*,*Chapalote(2)*,*Onaveño*,*Jala*,*Elotes Occidentales*,*Reventador*,*Gordo> Cristalino de Chihuahua*,*Tabloncillo*, *Maíz Dulce(Mex)*; Dente Branco(Ur)
**Central Mexico (g2)**	*Palomero Toluqueño*, *Cacahuacintle(2)*, *Chalqueño*, *Mushito*, *Harinoso de Ocho*, *Cónico(3)*, *Arrocillo Amarillo(2)*, *Cónico Norteño (Mx)*
**Southern Andes (g3)**	SC(1)**, Canario de Ocho (Ar);SC**(8), *Pisankalla*, *Ocho Corridas*,*Araucano (Ch); Canguil(2) (Ec); Confite Puneño*, *San Jeronimo (Pe); Cateto Sulino (Ur)*
** **	**G2—Mesoamerica lowland**
**Tropical lowland (g4)**	*Perola* (Bol); Cristalino Cubano *(*IsVir); *Amagaceño* (Col); *Salvadoreño*(2) (CR); *Cristalino Cubano* (2), Canilla, Chandelle (Cu); *Tusilla* (2)(Ec); *Caribeño Precoz* (Guad); *Quicheño precoz* (Guat); *Cateto Sulino* (Ur); *Cristalino Cubano*, *Tusón*(2) (Ve)
**South Mexico/Guatemala/Virgin IS/Venezuela (g5)**	Cristalino Costeño Tropical(IsVir); San Marceño, *Olotón*, *Quicheño Precoz*, *Comiteco*, Olotón > Nal-Tel, Negro de Chimaltenango, *Nal-Tel Blanco Tierra Baja* (Guat); *Zapalote Grande*, *Zapalote Chico* (3), *Nal-Tel>Harinoso*, *Tuxpeño*, *Tepecintle*, *Vandeno* (Mex); Sabanero (Ve)
**Lesser Antilles (g6)**	*Caribeño Precoz* (2) (IsVir); *Caribeño Precoz* (Mar); Cristalino Costeño Tropical (SVic)
**Greater and Lesser Antilles (g7)**	Cristalino Costeño Tropical (An); Cateto Nortista (Br), *Chandelle* (2) (RDom); *Chandelle* (2), *Cristalino Costeño Tropical* (2) (Guad); *Tusón* (Tri)
ng*	Cristalino Costeño Tropical (2), Tusón (Bar); Cristalino Cubano (Cu), Caribeño Precoz(2) (Guad); Salvadoreño-Clavillo (Pan); Cristalino Costeño Tropical (SnVic)
** **	**G3—South America- Andean Region**
**Bolivian highlands (g8)**	*Chechi*, *Hualtaco*, Chuspilla- Chuspillu, *Uchuquilla*, *Kulli*, *Hualtaco Colorado* (Bol); *Chulpi* (Ch); *Cuzco Cristalino Amarillo*, Chulpi, Kculli (Pe)
**Bolivian lowland (g9)**	*Blando Amazonico*, Pojoso Chico, *Acre Inter* (Bol); *Camelia* (Ch); Montaña (Ec)
**Columbian 1 (g10)**	*Pira Naranja*, *Yucatan*, *Guira*, *Pira*, *Andaqui*, *Sabanero*, *Negrito*, *Cariaco*, *Cacao* (Col)
**Columbian 2 (g11)**	*Cateto Assis* (Br); *Pollo*, Puya Grande, *Puya* (Col); *Cateto Sulino* (Ur)
**Highland Andean (g12)**	*Cateto Amarillo*, *Capia Blanca* (Ar); *Altiplano*, *Perola* (Bol); *Morocho* (2) (Ec); *San Jeronimo-Huancavelicano*, *Confite Morocho* (Pe)
**Ecuadorian highland (g13)**	*Racimo de Uva*, *Chillo*, Uchima, *Chulpi*, *Morocho*, Shima, *Mishca* (Ec); Cuzco (Pe)
**Central highland Andean (g14)**	*Culli* (Ar); Paru (Bol); *Kcello* (Ec); *Cuzcri*, *Mochero*, (Pe)
**Northern tropical lowland (g15)**	Pira, *Chococeño*, Montaña *(*Col), *Salpor*, *Nal-Tel Amarillo de Tierra Baja* (Guat); *Cariaco* (Ve)
**Moroti's (g16)**	*Coroico* (Bol); Cateto Grande (Br); Avati' Moroti' Ti', *Avati' Pichinga*, *Avati' Moroti'*, *Avati* (Py)
ng*	Aysuna (Bol); Duro Amazónico, (Bol); Ancashino, (Pe);
	**Populations not grouped**
	Cuartento Cateto, Cristal Sulino, Cateto (Ar); Uchuquilla (Bol), Cateto Paulista Grosso, Dente Paulista, Dente Riograndense, Dentado (Br); Amagaceño, Pira (2), Cabuya (Col); Chococeño (Ec); Negro de Chimaltenango, Nal-Tel Blanco Tierra Alta, Olotón, Quicheño Precoz, Negro de Altura-Negro de Tierra Fria, San Marceño (Guat); Bolita, Olotillo (Mex); Uchuquilla, Huancavelicano, Perla (Pe); Cuarentón-Cateto Colorado (Ur)

*ng, populations not grouped within of each main cluster

**SC, populations without classification into a landrace

Race names, (Number of accessions if there are more than one), and countries (An-Antigua, Ar-Argentina, Bar-Barbados, Bol-Bolivia, Br-Brasil, IsVir-Virgen Islands, Ch-Chile, Col-Colombia, CR-Costa Rica, Cu-Cuba, RDom- Dominican Republic, Ec-Ecuador, Guad- Guadeloupe Islands, Guat-Guatemala, Mar-Martinica, Mex-Mexico, Pan-Panama, Py-Paraguay, Pe-Peru, SVic-St Vicent, Tri-Trinidad and Tobago, Ur-Uruguay, Ven-Venezuela).The accessions labeled with italics represent the most representative accessions in the sub-structure analyses (arbitrary ancestor of cutoff > 80%).

The third main group, The South America–Andean region cluster (G3) included the rest of the South American populations and two populations from Guatemala. The South America–Andean region cluster (G3) encompassed nine sub-clusters and populations that did not group at this level: g8—mainly formed by landraces from Bolivian highlands but extended to Chile and Peru; g9 –lowland landraces from Bolivia; g10—Columbian sub-cluster; g11—second Columbian sub-cluster formed by Columbian, Brazilian and Uruguayan tropical populations; g12 –highland landraces from the Andes; g13 –landraces from the central highland Andes; g14 –typical landraces from the Ecuadorian highlands; g15 –northern tropical lowlands; and g16—Avati’ sub-cluster.

### Genetic diversity in Latin American maize germplasm

Genetic diversity values, average number of alleles per locus, and unique alleles found between the three main groups and sub-groups identified by Structure and sub-structure analyses for the 194 maize populations are presented in Table 3. Overall genetic diversity for all 194 accessions was 0.62, all markers were polymorphic, and 291 alleles were detected for the 28 SSRs (ranging from 2–21 per locus with an average of 10.39). The largest number of unique alleles (24) per main group was obtained in the Andean maize (G3); other main clusters had 15 (G1) to 14 (G2) alleles. The highest diversity index was found within G1 (0.63) and the lowest within G3 (0.57). At the sub-cluster level, the largest number of alleles (192) was found in the subgroup g1 formed by populations from northern Mexico, and the mixed subgroup g5 had the largest number of unique alleles (21). In the genetic distance matrix for 194 total populations (data not shown), the maximum genetic distance value (0.73) was found between the Early Caribbean population from the Virgin Islands (from G2) and Pisankalla Chile’s lowland landrace from G1. The minimum genetic distance (0.18) was found between accessions from the highlands of the Andes (both within g12 of G3).

**Table 3:** Genetic diversity summary statistics based on 28 microsatellite markers (SSRs) determined for clusters assigned by structure and sub-structure analysis.

Groups	sub-groups	Number of populations	Alleles	Unique Alleles	Genetic Diversity
**G1**		46	227	15*	0.63
	**g1**	17	192	24**	0.60
	**g2**	12	176	11**	0.61
	**g3**	17	173	15**	0.61
**G2**	** **	58	221	14*	0.59
	**g4**	17	172	10**	0.56
	**g5**	18	191	21**	0.58
	**g6**	4	112	5**	0.47
	**g7**	11	150	4**	0.56
	**none**	8	145	1**	0.57
**G3**		65	226	24*	0.57
	**g8**	10	127	4**	0.46
	**g9**	6	114	2**	0.49
	**g10**	9	143	6**	0.56
	**g11**	5	115	3**	0.55
	**g12**	8	127	8**	0.48
	**g13**	8	117	5**	0.47
	**g14**	5	120	3**	0.52
	**g15**	6	119	3**	0.53
	**g16**	6	114	2**	0.49
	**none**	3	101	1**	0.45
**Mixed populations**		25	202	10*	0.61
**Total**		194	291	Not applicable	0.62

* Unique alleles compared between clusters

** unique alleles compared between sub-clusters of the same cluster

Genetic diversity and alleles based on average measurements for each cluster and sub-cluster. Arbitrary ancestry contribution cutoff of 51%.

## Discussion

### Mexican maize racial complexes

In the latest published classification [23], 59 distinct Mexican landraces were reported; classification based on the SSRs run on the 38 bulked Mexican populations in the current study corresponded to 27 of them. These populations clustered into distinct racial complexes consistent with past studies and geographical distribution. Linking ancestral maize accessions to cultures that selected and grew them prior to (or even after) the arrival of the Spaniards is complicated by a lack of dates for specific landraces in the archeological record, despite all we know about when and where the different cultures flourished. However, charred corncobs found in the ancient city of Teotihuacan near present day Mexico City suggest that many races cultivated in the first century A.D are similar to modern maize landraces found in the Central Valleys of Mexico [45,46].

The distinct racial complexes obtained for Palomero Toluqueño and Arrocillo Amarillo (Central Valleys), Chapalote (Sierra Madre Occidental), and Nal-Tel (Southern and Southwestern) are consistent with differences between the Ancient Indigenous landraces as a consequence of their independent development in different locations and environments. These ancient landraces are believed to have originated in Mexico from tunicate primitive maize, and have been in existence for a very long time [25]. In contrast, the Modern Incipient landraces (Bolita, Celaya, Chalqueño and Conico Norteño) have evolved since (and often as a consequence of) the arrival of the Spaniards, and have not yet reached conditions of racial uniformity.

### Genetic diversity, structure and sub-structure patterns in Latin-American germplasm

Genetic diversity levels in populations from the maize center of origin were high, and decreased as distance from the center of origin grew, reaching a low in South American populations, in agreement with a previous study [14]. High overall genetic diversity, as well as a high number of unique alleles found within sub-groups, supports the great diversity present within the native races of maize in Latin America. The strong separation observed in the NJ cluster, PCoA, and structure analyses between South America–Andean region maize (G3) with the Mexico and Southern Andes group (G1) and Mesoamerica Lowland (G2) indicates that only a part of the germplasm from G1 and G2 has contributed to the Andean region. Presumably this portion of the genetic contribution could be linked to the maize expansion centered on highlands agriculture system from Mesoamerica through the Panama highlands into the Andean regions proposed in previous studies [3, 47].

In spite of the differences in methodology used for determining the patterns of population structure for the most representative accessions from the Americas in a previous study that included a subset of the data from the current study [17], the two studies found the same three main cluster model, consistent with earlier studies [3,16]. The sub-structure model was able to detect more specific sub-groups with solid geographic patterns, including elevation-dependent patterns, at country and regional levels. The most representative cases were: the Mexican highlands main cluster documented in the global diffusion of maize study [17], which was partitioned into sub-groups in the current study, and were named Northern Mexico (g1) and Central Mexico (g2); the Andes cluster, which was subdivided into the Bolivian highlands (g8), Highland Andes (g12), Ecuadorian Highland (g13), and Central Highland Andes (g14); the Middle South-America main cluster, which was partitioned into Bolivian lowland (g9), Moroti’ (g16) and Columbian2 (g11); and the Northern US Flints main cluster, which is mirrored here by a southern Andes subgroup (g4; the relationships between US and South American accessions are explained below). Accessions in common between the two studies, including naming codes and corresponding details are given in S4 Table.

### Relationships between ancient and modern maize inferred by NJ cluster and sub-structure analyses

Maize spread into the southwestern US from northern Mexico, and finally into the northern US and Canada from the southwestern US [3]. In agreement with morphological [48] and genetic similarities [1,16,49], pre-Columbian interchange of ancient popcorns from North and South America was inferred by the presence of ancient South American popcorns (Canguil, Confite Puneño and Pisankalla) in the Mexico and Southern Andes group (G1). This group also represents post-Columbian maize introductions from the US to southern South America by the incidence of US-derived races like Araucano, Ocho Corridas, and Dente Branco [50,51].

**Fig 7:**
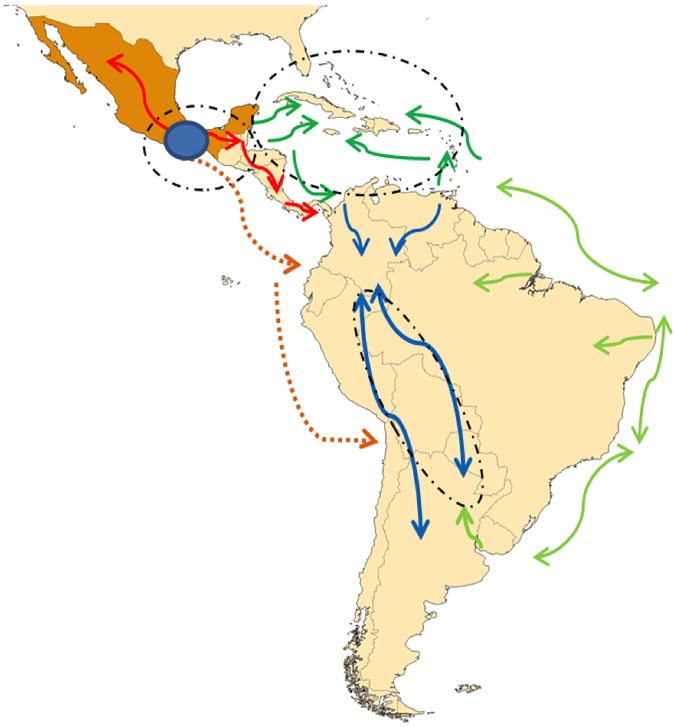
Suggested maize migration routes from its center of origin in Mesoamerica based on archeological evidence, historic and anthropological studies, and genetic relationships. Red arrows indicate early maize dispersal from its origin center in Mesoamerica towards northern Mexico and Central America; dashed orange arrows represents the likely Pacific Ocean routes via maritime technologies in Pre-Columbian times; green arrows show maize migrations from the mainland to the Caribbean; light green arrows show routes followed by the Caribbean communities along the eastern coast and rivers; blue arrows correspond to movements in the Andean region in different directions. Ovals correspond to important zones of maize germplasm interchange.

The Mesoamerica lowland group (G2) sheds light on the origin of Caribbean maize, supporting the theory that maize was introduced into the Caribbean twice [8]: first from Venezuela via the southernmost Caribbean islands, and secondly from Central America (Panama, Costa Rica and southern Mexico), (Fig 7). We cannot confirm that these introductions are linked with specific migrations of people, but they certainly could be related to two separate human migration events, one from South America around 5500 BC, and the other from the Yucatan in Central America, beginning around 3000–4000 BC. [52]. Considering the date of domestication of maize, however, very early human migrations were not carrying maize as it is now known; if it was carried out of the center of origin, it was in a very primitive form. Regardless, maize carried out from the two population centers may have then experienced a fairly complete mixing due to: i) the lack of geographical barriers, allowing free migration of people between these islands (presumably, accompanied by maize); and ii) massive human migrations beginning after the arrival of the Spaniards, who moved people (and food) within and between the New and Old World continents, using the Caribbean as a crossing point [26]. The genetic relationships between Caribbean maize and maize from mainland South American seen in the current study may also be the result of the historically documented movement of the Taino-Arawak, people of the Caribbean, into the lowlands of Peru, Brazil and Bolivia via rivers to reach the foothills of the Andes [53]. These human migrations may have resulted in the germplasm of the Northern Territory cluster [9] and in the current data as well.

The South America–Andean group (G3) displayed an overlapping area of different sub-clusters especially in the Central Andes, supporting the hypothesis that this region was an important center of maize interchange or meeting ground [3,54]. However, the origin of maize in the Andean highlands is still unclear, as a direct connection between the Andean highlands and the highlands of Guatemala was not found in our study, or in a previous study [3]. It could be that the average altitude difference between our Andean highlands populations (from ~ 2,400 m) and Mesoamerica highlands (~ 2,000 m) blurs a direct connection between Mesoamerica and Andean maize. In addition, indigenous South American landraces associated with Mesoamerican landraces may hint at an ancient relationship, but the antiquity of introductions makes it difficult to follow direct links between them. New archaeological evidence could support the previous existence of extinct races of maize which would clarify relationships between progeny of primitive maize lineages. The intermediate placement of some races from northern South America in the PCoA may have been caused by incomplete sampling, due possibly to the extinction of some races of maize prior to collection, thus not allowing all races to cluster with their closest relatives. This may further explain why so many studies have trouble identifying the origin of Andean maize. The arrival of the Spaniards may have directly or indirectly interfered with existing production systems. Land planted to maize may have more recently decreased with the introduction of high-value commercial crops like sugarcane and wheat. Equally, the drastic decrease in human native population levels immediately after the discovery of the New World by Europeans caused by the introduction of new human diseases would have undoubtedly affected the maintenance of maize races in those regions most impacted.

Although mutation and selection over such long time periods as those suggested for domesticated maize evolution [7] make it difficult to recognize the links between ancient landraces, Structure and NJ cluster analyses that find Central American populations frequently interspersed with other groups may reflect patterns of independent migrations through Central America at different times. In this sense, two distinct maize expansions from Mesoamerica may be proposed and cross validated using maize genetic data [47]. The first, from Central America through the highlands into the Andes and the western coast of South America, faces geographical barriers including extremely steep mountains in Central America, and the jungles and mountains of Colombia. This route is somewhat unlikely, especially since maize cannot quickly adapt to large differences in elevation [55], and indeed, our results do not offer strong support for this maize migration route. The second route, a lowland expansion from coastal Panama along the northeast of South America, is supported by the Mesoamerica lowland group that encompasses eastern South America maize populations in our study. The maximum genetic distance between Pisankalla and Early Caribbean landraces suggests two independent migration patterns for different lowland maize lineages, probably via both the western and the eastern Latin American coasts. These paths also match with the migration patterns of human American populations [56]. Migration along the coasts would have been easier, and pre-Columbian maritime navigation connecting Mesoamerica and South America has been reported [57]. The map in Fig 7 shows the suggested maize migration routes in pre- and post-Columbian America from the center of maize origin in Mesoamerica, consistent with the genetic relationships found in this study, archeological evidence, and historic and anthropological studies.

Maize has been a cornerstone in past and current cultures throughout the Americas, which has led to the development and continuous improvement of many landraces. Based on the association relationships found across and within groups of the accessions evaluated in this study, it is evident that the structure of Latin American maize genetic pools are dynamic and influenced by discrete micro- and macro-environmental zones, human migrations and trade, as well as landrace selection and conservation by indigenous communities. Our results have a practical application, for example facilitating the identification of gene bank pools and accessions containing valuable, unique alleles for breeding, providing access to allelic diversity that has been eliminated locally due to selection pressure and evolution of populations in the target environments inherent to human migration and settlement.

## Supporting information

S1 Fig Racial relationships of the corn of Mexico. Landraces within big cells correspond to the definitive racial complex system of classification of Goodman and Brown [1]; the landraces within small cells correspond to the groups/ sub-groups documented in a more recent classifation [2].
(DOCX)

S2 Fig Mexico. Plots of the log likelihood **(a)** and Δ*K* **(b)** for 38 Mexican accessions from structure analysis. For the log likelihood plots and the calculation of *ΔK*, the average log likelihood from among the five replicate runs performed at each *K* is plotted (except for *K* = 1, where only one run was performed). The high values of *ΔK* (2, 4 and 6) are labeled with red. *K* = 4 was selected like the optimal structure model.
(DOCX)

S3 Fig Latin America and the Caribbean. Plots of the log likelihood **(a)** and Δ*K* **(b)** for 194 Latin America and the Caribbean accessions, including Mexican accessions from structure analysis. For the log likelihood plots and the calculation of *ΔK*, the average log likelihood from among the five replicate runs performed at each *K* is plotted (except for *K* = 1, where only one run was performed). The high values of *ΔK* (2 and 3) are labeled with red.
(DOCX)

S4 Fig Mexico and southern Andes cluster (G1). Plots of the log likelihood **(a)** and Δ*K* **(b)** for 48 Mexico and southern Andes accessions from substructure analyses. For the log likelihood plots and the calculation of *ΔK*, the average log likelihood from among the five replicate runs performed at each *K* is plotted (except for *K* = 1, where only one run was performed). The high values of *ΔK* (2 and 3) are labeled with red. The *K* = 3 was selected like the optimal substructure model.
(DOCX)

S5 Fig Mesoamerica lowland cluster (G2). Plots of the log likelihood **(a)** and *ΔK* **(b)** for 58 accessions from Mexico, Central America, the Caribbean and northeastern of South America. For the log likelihood plots and the calculation of *ΔK*, the average log likelihood from among the five replicate runs performed at each *K* is plotted (except for *K* = 1, where only one run was performed). The high values of *ΔK* (2 and 4) are labeled with red. The *K* = 4 was selected like the optimal substructure model.
(DOCX)

S6 Fig South America-Andean Region (G3). Plots of the log likelihood **(a)** and *ΔK* **(b)** for 64 accessions from South America- Andean region. For the log likelihood plots and the calculation of *ΔK*, the average log likelihood from among the five replicate runs performed at each *K* is plotted (except for *K* = 1, where only one run was performed). The high values of *ΔK* (2 and 9) are labeled with red. The *K* = 9 was selected like the optimal substructure model.
(DOCX)

S1 Table Passport data for the 194 entries in the study including accession abbreviation, race, country, and location of collection. (DOCX)

S2 Table List of the 28 SSR loci used to characterize the landraces in this study. ^a^ SSR location in the genome. ^b^ SSR repeat unit. ^c^ Allele size range in bp over the whole dataset. Loci^***§***^ in common with global diffusion of maize study [7].
(DOCX)

S3 Table Mexican race names, accession, and altitude of the regions where the accessions were grown. **Classification of accessions according to 5 previous studies.** ᵃResults of this study based on the structure analysis for *K* = 4, group 1 corresponds to maize accessions from Sierra Madre Occidental, group 2 to Southern Mexico, group 3 to Central Valleys, and group 4 to Chapalote Complex; ᵇResults of this study based on Neighbor Joining (NJ) cluster analysis; ᶜRacial Complexes, classification based on a combination of morphological, cytological and isozyme data [1]; ᵈClassification based on microsatellite data [8]; ᵉClassification based on a combination of morphological and isozyme data [9]; ᶠClassification based on cob morphological characteristics [10]; ᶢClassification based on morphological data [11]; ng: race not clustered to a specific group, np: race not included in the study.
(DOCX)

S4 Table Accessions in common with the study: Out of America: tracing the genetic footprints of the global diffusion of maize [7]. Codes used and correspondences between clusters inferred with the most representative accessions with the study mentioned, and structure and sub-structure patterns in the present study, ng populations not grouped at structure or sub-structure level.
(DOCX)

S5 Table Allelic frequencies for the 194 maize populations and SSRs markers used in the genotypic characterization. Freqsbulk_194pops data correspond to allelic frequency of each allele in the bulks, bulk number correspond in the same order to accesion number on the passport data information provided. Freqsindivi_2010 sheet correspond to the simulated individual alleles for each bulk.
(XLSX)
