# Supplementary material for: Genetic diversity and population structure of native maize populations in Latin America and the Caribbean
Source: PLoS One. 2017 Apr 12;12(4):e0173488. doi: 10.1371/journal.pone.0173488 (PMC5389613; doi:10.1371/journal.pone.0173488)
Supplement: S1 Fig — Landraces within big cells correspond to the definitive racial complex system of classification of Goodman and Brown [1]; the landraces within small cells correspond to the groups/ sub-groups documented in a more recent classifation [2]. (DOCX) [file pone.0173488.s001.docx]

**Fig. S1: Racial relationships of the corn of Mexico.** Landraces within big cells correspond to the definitive racial complex system of classification of Goodman and Brown [26]; the landraces within small cells correspond to the groups/ sub-groups documented in a more recent classifation [39].
