# Supplementary material for: Genetic diversity and population structure of native maize populations in Latin America and the Caribbean
Source: PLoS One. 2017 Apr 12;12(4):e0173488. doi: 10.1371/journal.pone.0173488 (PMC5389613; doi:10.1371/journal.pone.0173488)
Supplement: S2 Fig — Plots of the log likelihood (a) and ΔK (b) for 38 Mexican accessions from structure analysis. For the log likelihood plots and the calculation of ΔK, the average log likelihood from among the five replicate runs performed at each K is plotted (except for K = 1, where only one run was performed). The high values of ΔK (2, 4 and 6) are labeled with red. K = 4 was selected like the optimal structure model. (DOCX) [file pone.0173488.s002.docx]

**Figure S2: Mexico**

Plots of the log likelihood **(a)** and Δ*K* **(b)** for 38 Mexican accessions from structure analysis. For the log likelihood plots and the calculation of *ΔK,* the average log likelihood from among the five replicate runs performed at each *K* is plotted (except for *K* = 1, where only one run was performed). The high values of *ΔK* (2, 4 and 6) are labeled with red. *K*=4 was selected like the optimal structure model.
