# Supplementary material for: Genetic diversity and population structure of native maize populations in Latin America and the Caribbean
Source: PLoS One. 2017 Apr 12;12(4):e0173488. doi: 10.1371/journal.pone.0173488 (PMC5389613; doi:10.1371/journal.pone.0173488)
Supplement: S3 Fig — Plots of the log likelihood (a) and ΔK (b) for 194 Latin America and the Caribbean accessions, including Mexican accessions from structure analysis. For the log likelihood plots and the calculation of ΔK, the average log likelihood from among the five replicate runs performed at each K is plotted (except for K = 1, where only one run was performed). The high values of ΔK (2 and 3) are labeled with red. (DOCX) [file pone.0173488.s003.docx]

**Figure S3 Latin America and the Caribbean**

Plots of the log likelihood **(a)** and Δ*K* **(b)** for 194 Latin America and the Caribbean accessions, including Mexican accessions from structure analysis. For the log likelihood plots and the calculation of *ΔK,* the average log likelihood from among the five replicate runs performed at each *K* is plotted (except for *K* = 1, where only one run was performed). The high values of *ΔK* (2 and 3) are labeled with red.

The high values of *ΔK* were analyzed*.* The admixed accessions identified at *K*=2 were mainly from a complex group that contains populations from Mexico, Central America, Caribbean and some populations from South America lowlands, and a consistent Andean group. At *K*=3, the complex group was split into Mexico and southern Andes group, and Mesoamerica lowland group (including populations from the Caribbean and Central America, and populations from South America lowlands); the Andean group remained consistent.

Taking an arbitrary ancestry proportion greater than 51.0%, for *K*=2 and *K*=3, 98 % and 87 % of the total accessions were assigned respectively to each group. The *K*=3 was selected like the optimal structure model.
