# Supplementary material for: Genetic diversity and population structure of native maize populations in Latin America and the Caribbean
Source: PLoS One. 2017 Apr 12;12(4):e0173488. doi: 10.1371/journal.pone.0173488 (PMC5389613; doi:10.1371/journal.pone.0173488)
Supplement: S5 Fig — Plots of the log likelihood (a) and ΔK (b) for 58 accessions from Mexico, Central America, the Caribbean and northeastern of South America. For the log likelihood plots and the calculation of ΔK, the average log likelihood from among the five replicate runs performed at each K is plotted (except for K = 1, where only one run was performed). The high values of ΔK (2 and 4) are labeled with red. The K = 4 was selected like the optimal substructure model. (DOCX) [file pone.0173488.s005.docx]

**Figure S5: Mesoamerica lowland cluster (G2).** Plots of the log likelihood **(a)** and *ΔK* **(b)** for 58 accessions from Mexico, Central America, the Caribbean and northeastern of South America. For the log likelihood plots and the calculation of *ΔK,* the average log likelihood from among the five replicate runs performed at each *K* is plotted (except for *K* = 1, where only one run was performed). The high values of *ΔK* (2 and 4) are labeled with red. The *K*=4 was selected like the optimal substructure model.
