# Supplementary material for: Genetic diversity and population structure of native maize populations in Latin America and the Caribbean
Source: PLoS One. 2017 Apr 12;12(4):e0173488. doi: 10.1371/journal.pone.0173488 (PMC5389613; doi:10.1371/journal.pone.0173488)
Supplement: S6 Fig — Plots of the log likelihood (a) and ΔK (b) for 64 accessions from South America- Andean region. For the log likelihood plots and the calculation of ΔK, the average log likelihood from among the five replicate runs performed at each K is plotted (except for K = 1, where only one run was performed). The high values of ΔK (2 and 9) are labeled with red. The K = 9 was selected like the optimal substructure model. (DOCX) [file pone.0173488.s006.docx]

**Figure S6: South America-Andean Region (G3).** Plots of the log likelihood **(a)** and *ΔK* **(b)** for 64 accessions from South America- Andean region. For the log likelihood plots and the calculation of *ΔK,* the average log likelihood from among the five replicate runs performed at each *K* is plotted (except for *K* = 1, where only one run was performed). The high values of *ΔK* (2 and 9) are labeled with red. The *K*=9 was selected like the optimal substructure model**.**

In NJ cluster analysis, as well as structure and substructure analyses, some races were placed in more than one cluster/subcluster or appeared to be admixed. For example, accessions from Perola, Amagaceño were placed in more than one cluster. Furthermore, The Caribbean maize classification is more complex, for example, Coastal Tropical Flint race accessions were placed in three subclusters and also appeared to be admixed. These divergences in some cases may indicate that the allocation of a population to a subgroup does not reflect the names of the races that have been classified, reflecting a limitation in the classification based on morphological parameters. In addition, comparisons between regions are often hampered by a lack of uniformity in the race names and inconsistencies in the conceptual definition of race [3,54]
