# Supplementary material for: Genetic diversity and population structure of native maize populations in Latin America and the Caribbean
Source: PLoS One. 2017 Apr 12;12(4):e0173488. doi: 10.1371/journal.pone.0173488 (PMC5389613; doi:10.1371/journal.pone.0173488)
Supplement: S1 Table — (DOCX) [file pone.0173488.s007.docx]

**Table S1: Passport data for the 194 entries in the study including accession abbreviation, race, country, and location of collection.**

| **ID** | **Accession** | **Races** | **Country** | **Altitude** | **Coordinates** | |
| --- | --- | --- | --- | --- | --- | --- |
|  |  |  |  | **(m.a.s.l)** |  |  |
| 1 | ***ANTI GP2*** | Coastal Tropical Flint | Antigua | 40 | 17.10 | -61.77 |
| 2 | ***ARGE 486*** | Cuarento Cateto | Argentina | 300 | -34.900 | -57.950 |
| 3 | ***ARGE 564*** | Cateto Amarillo | Argentina | 1700 | -26.05 | -65.83 |
| 4 | ***ARGE GP8*** | Cristal Sulino | Argentina | 1554 | -29.000 | -62.000 |
| 5 | ***PPS920*** |  | Argentina | 81 | -34.800 | -61.667 |
| 6 | ***ARGENT 24*** | Canario de Ocho | Argentina | 117 | -36.156 | -63.506 |
| 7 | ***ARGENT 306*** | Cateto | Argentina | 1006 | -34.5 | -68.5 |
| 8 | ***ARGENT TUC43*** | Culli | Argentina | 2500 | -23.2 | -65.35 |
| 9 | ***ARZM 03 013*** | Camelia | Argentina | 62 | -30.82 | -57.98 |
| 10 | ***ARGENT TUC16*** | Capia Blanca | Argentina | 2500 | -23.2 | -65.35 |
| 11 | ***BARB 5*** | Coastal Tropical Flint | Barbados | 100 | 13.26 | -59.61 |
| 12 | ***BARB GP2*** | Tusón | Barbados | 70 | 13.12 | -59.60 |
| 13 | ***BARB GP1*** | Coastal Tropical Flint | Barbados | 100 | 13.18 | -59.58 |
| 14 | ***BOLI 905*** | Altiplano | Bolivia | 1976 | -20.00 | -64.20 |
| 15 | ***BOLI 929*** | Perola | Bolivia | 1828 | -17.80 | -63.17 |
| 16 | ***BOZMO 214*** | Uchuquilla | Bolivia | 2700 | -17.60 | -66.03 |
| 17 | ***BOZM 1609*** | Blando Amázonico | Bolivia | 537 | -18.139 | -60.019 |
| 18 | ***BOLI 711*** | Perola | Bolivia | 475 | -21.52 | -64.75 |
| 19 | ***BOLIVI 957*** | Chuspilla, Chuspillu | Bolivia | 1947 | -20.00 | -64.20 |
| 20 | ***BOLIVI 1037*** | Coroico | Bolivia | 156 | -11.33 | -67.67 |
| 21 | ***BOLIVI 928*** | Checchi | Bolivia | 1976 | -20.00 | -64.20 |
| 22 | ***BOLIVI 968*** | Aysuma | Bolivia | 1947 | -20.00 | -64.20 |
| 23 | ***BOZM 1791*** | Hualtaco | Bolivia | 242 | -16.992 | -65.152 |
| 24 | ***BOZM 988*** | Hualtaco Colorado | Bolivia | 2900 | -21.47 | -65.35 |
| 25 | ***BOLIVI 351*** | Pojoso Chico, Ecuatoriana | Bolivia | 1050 | -19.65 | -63.68 |
| 26 | ***BOZM 1681*** | Duro Amazónico | Bolivia | 418 | -17.775 | -63.194 |
| 27 | ***BOLIVI 90*** | Acre Interlock | Bolivia | 500 | -17.85 | -60.783 |
| 28 | ***BOZM 694*** | Uchuquilla | Bolivia | 2600 | -21.87 | -64.15 |
| 29 | ***BOZM 677*** | Paru | Bolivia | 3000 | -17.083 | -66.85 |
| 30 | ***BOZM 96*** | Kulli | Bolivia | 2150 | -18.18 | -65.00 |
| 31 | ***BRAZIL 2441*** | Cateto Paulista Grosso (Cateto Grosso) | Brasil | 1081 | -15.78 | -47.936 |
| 32 | ***BRAZIL 1546*** | Cateto Assis Brasil | Brasil | 274 | -29.333 | -54.167 |
| 33 | ***BRAZIL 2305*** | Dente Paulista | Brasil | 770 | -23.33 | -46.38 |
| 34 | ***BRAZIL 953*** | Dente Riograndense Rugoso | Brasil | 274 | -28.183 | -54.733 |
| 35 | ***BRAZIL PE012*** | Dentado | Brasil | 553 | -8.283 | -35.967 |
| 36 | ***26373*** | Cateto Nortista | Brasil | 801 | -16.667 | -49.255 |
| 37 | ***BRAZIL 60*** | Cateto Grande | Brasil | 500 | -22.217 | -54.8 |
| 38 | ***BRVI 104*** | Coastal Tropical Flint | Virgen Islands | 16 | 18.443 | -64.582 |
| 39 | ***BRVI 117*** | Cuban Flint | Virgen Islands | 190 | 18.419 | -64.629 |
| 40 | ***BRVI 139*** | Early Caribbean | Virgen Islands | 63 | 18.395 | -64.647 |
| 41 | ***BRVI 142*** | Early Caribbean | Virgen Islands | 92 | 18.419 | -64.662 |
| 42 | ***CHZM 13 080*** | Camelia | Chile | 625 | -33.45 | -70.667 |
| 43 | ***PPS1066*** |  | Chile | 234 | -40.917 | -73.167 |
| 44 | ***PPS938*** |  | Chile | 458 | -38.850 | -72.367 |
| 45 | ***PPS941*** |  | Chile | 458 | -39.517 | -71.867 |
| 46 | ***PPS949*** |  | Chile | 338 | -39.583 | -72.033 |
| 47 | ***PPS961*** |  | Chile | 338 | -40.233 | -72.017 |
| 48 | ***PPS939*** |  | Chile | 458 | -38.850 | -71.700 |
| 49 | ***CHZM 08 060*** | Pisankalla | Chile | 450 | -37.75 | -72 |
| 50 | ***PPS55*** |  | Chile | 337 | -40.633 | -72.317 |
| 51 | ***CHZM 09 030*** | Araucano | Chile | 100 | -38.733 | -72.95 |
| 52 | ***CHZM 01 062*** | Chulpi | Chile | 2300 | -19.3 | -69.26 |
| 53 | ***CHZM 08 049*** | Ocho Corridas | Chile | 250 | -37.45 | -72.00 |
| 54 | ***NARINO 369*** | Pija naranja | Colombia | 1554 | 1.51 | -77.14 |
| 55 | ***NARINO 392*** | Chococeno | Colombia | 2000 | 1.3 | -78.3 |
| 56 | ***TOLIMA 389*** | Yucatan | Colombia | 450 | 3.45 | -75.15 |
| 57 | ***MAGDAL 443*** | Guirua | Colombia | 1860 | 10.00 | -74.00 |
| 58 | ***CAUCA 384*** | Amagaceno | Colombia | 1600 | 3.055 | -76.211 |
| 59 | ***ANTI 392*** | Amagaceño | Colombia | 1371 | 6.55 | -75.83 |
| 60 | ***BOYACA 462*** | Pira | Colombia | 900 | 5.30 | -73.30 |
| 61 | ***CUNDIN 327*** | Pira | Colombia | 2377 | 5.317 | -73.817 |
| 62 | ***TOLIMA 390*** | Pira | Colombia | 312 | 4.033 | -74.967 |
| 63 | ***CAQUET 321*** | Andaqui | Colombia | 700 | 1.00 | -74.00 |
| 64 | ***CUNDIN 428*** | Sabanero | Colombia | 1960 | 4.528 | -73.929 |
| 65 | ***CUNDIN 465*** | Pollo | Colombia | 1600 | 5.00 | -74.00 |
| 66 | ***COLOMB 613*** | Negrito | Colombia | 540 | 11.25 | -74.18 |
| 67 | ***SANTAN 332*** | Puya grande | Colombia | 978 | 7.61 | -72.65 |
| 68 | ***CUNDIN 480*** | Pira | Colombia | 1000 | 5.00 | -74.00 |
| 69 | ***NARINO 534*** | Montana | Colombia | 1500 | 1.3 | -78.3 |
| 70 | ***SANTAN 317*** | Cabuya | Colombia | 1920 | 7.383 | -72.65 |
| 71 | ***CORDOB 342*** | Cariaco | Colombia | 229 | 8.717 | -75.883 |
| 72 | ***MAGDAL 390*** | Puya | Colombia | 229 | 8.317 | -73.633 |
| 73 | ***SANTAS 340*** | Cacao | Colombia | 1252 | 6.283 | -73.167 |
| 74 | ***CRIC 141*** | Salvadoreño>Oloton | Costa Rica | 50 | 10.89 | -85.01 |
| 75 | ***CRIC 166*** | Salvadoreño | Costa Rica | 80 | 10.25 | -85.62 |
| 76 | ***CUBA 44*** | Cuban Flint | Cuba | 244 | 20.383 | -76.433 |
| 77 | ***CUBA 54*** | Canilla | Cuba | 305 | 20.90 | -76.25 |
| 78 | ***CUBA 63*** | Cuban Flint | Cuba | 91 | 20.667 | -75.667 |
| 79 | ***CUBA 85*** | Chandelle | Cuba | 80 | 20.30 | -76.25 |
| 80 | ***CUBA 12*** | Cuban Flint | Cuba | 122 | 22.417 | -83.733 |
| 81 | ***RDOM 270*** | Chandelle | Dominican Republic | 130 | 19.73 | -70.92 |
| 82 | ***RDOM GP1*** | Chandelle | Dominican Republic | 170 | 19.11 | -70.30 |
| 83 | ***ECUADO 398*** | Racimo de Uva | Ecuador | 2423 | -0.083 | -78.417 |
| 84 | ***ECUA 476*** | Morocho | Ecuador | 2195 | -0.050 | -78.450 |
| 85 | ***ECUA 617*** | Tusilla | Ecuador | 549 | -4.15 | -78.92 |
| 86 | ***ECUA 500*** | Canguil | Ecuador | 2213 | 0.167 | -78.417 |
| 87 | ***ECUA 696*** | Canguil | Ecuador | 1920 | 0.22 | -78.25 |
| 88 | ***ECUADO 486*** | Chillo | Ecuador | 2560 | -1.733 | -78.583 |
| 89 | ***ECUADO 704*** | Kcello Ecuatoriano | Ecuador | 1800 | -4.10 | -79.30 |
| 90 | ***ECUA 418*** | Morocho | Ecuador | 2195 | -0.317 | -78.45 |
| 91 | ***ECUA 881*** | Tusilla | Ecuador | 1372 | -3.00 | -79.00 |
| 92 | ***ECUADO 573*** | Montana | Ecuador | 2259 | 0.22 | -78.25 |
| 93 | ***ECUADO 746*** | Uchima | Ecuador | 1737 | -4.167 | -79.5 |
| 94 | ***ECUADO 979*** | Chococeno | Ecuador | 27 | 0.40 | -79.30 |
| 95 | ***ECUADO 424*** | Chulpi | Ecuador | 2579 | 0.050 | -78.200 |
| 96 | ***ECUADO 418*** | Morocho | Ecuador | 2195 | -0.317 | -78.45 |
| 97 | ***ECUADO X14237*** | Shima | Ecuador | 2689 | -3.09 | -79.01 |
| 98 | ***ECUADO 459*** | Mishca | Ecuador | 2286 | -0.111 | -78.291 |
| 99 | ***DES103*** | Early Caribbean | Guadeloupe Islands | 40 | 16.308 | -61.073 |
| 100 | ***GFO053*** | chandelle | Guadeloupe Islands | 40 | 16.226 | -61.393 |
| 101 | ***GFO059*** | Coastal Tropical Flint | Guadeloupe Islands | 40 | 16.251 | -61.529 |
| 102 | ***GUAD 6*** | Early Caribbean | Guadeloupe Islands | 100 | 16.317 | -61.367 |
| 103 | ***STB073*** | Chandelle | Guadeloupe Islands | 286 | 16.026 | -61.723 |
| 104 | ***EGT014*** | Coastal Tropical Flint | Guadeloupe Islands | 40 | 16.275 | -61.257 |
| 105 | ***MGA201*** | Early carribean | Guadeloupe Islands | 143 | 15.903 | -61.249 |
| 106 | ***DES106*** | Early Caribbean | Guadeloupe Islands | 40 | 16.334 | -61.015 |
| 107 | ***MGA235*** | Early Caribbean | Guadeloupe Islands | 10 | 15.955 | -61.312 |
| 108 | ***GUAT 162*** | Quicheño early | Guatemala | 1753 | 15.09 | -91.15 |
| 109 | ***GUAT 36*** | San Marceño | Guatemala | 1829 | 15.41 | -91.15 |
| 110 | ***GUAT 45*** | Olotón | Guatemala | 1749 | 14.633 | -90.517 |
| 111 | ***GUAT 527*** | Quicheño early | Guatemala | 1844 | 15.50 | -91.77 |
| 112 | ***GUAT 529*** | Comiteco | Guatemala | 1600 | 15.42 | -91.77 |
| 113 | ***GUAT 606*** | Olotón > Nal-Tel | Guatemala | 1890 | 15.28 | -91.43 |
| 114 | ***GUAT 820*** | Negro de Chimaltenango | Guatemala | 1219 | 15.40 | -90.38 |
| 115 | ***GUATEM 685*** | Negro de Chimaltenango | Guatemala | 1219 | 15.40 | -90.38 |
| 116 | ***GUATEM SH22*** | Olotón | Guatemala | 1749 | 14.633 | -90.517 |
| 117 | ***GUATEM 213*** | Quicheno Early | Guatemala | 610 | 15.01 | -91.11 |
| 118 | ***GUATEM 390*** | Negro de Altura (Negro de Tierra Fria) | Guatemala | 2316 | 14.80 | -91.52 |
| 119 | ***GUATEM 404*** | Nal-Tel Blanco Tierra Alta | Guatemala | 2774 | 15.05 | -91.41 |
| 120 | ***GUATEM 155*** | Nal-Tel Blanco Tierra Baja | Guatemala | 1006 | 15.09 | -90.49 |
| 121 | ***GUATEM 548*** | Salpor | Guatemala | 688 | 14.62 | -91.60 |
| 122 | ***GUATEM 10*** | San Marceno | Guatemala | 206 | 14.85 | -92.08 |
| 123 | ***GUATEM 93*** | Nal-Tel Amarillo de Tierra Baja | Guatemala | 914 | 14.783 | -89.55 |
| 124 | ***MART 4*** | Early Caribbean | Martinica | 213 | 14.75 | -61.18 |
| 125 | ***CHIH 131*** | Gordo | Mexico | 2095 | 29.20 | -108.12 |
| 126 | ***CHIH 133*** | Azul > Cristalino de Chihuahua | Mexico | 2095 | 29.20 | -108.15 |
| 127 | ***CHIH 150*** | Palomero Toluqueño > Cristalino de Chihuahua | Mexico | 2140 | 29.37 | -107.73 |
| 128 | ***CHIH 207*** | Cristalino de Chihuahua>Apachito | Mexico | 2510 | 26.80 | -107.25 |
| 129 | ***CHIH 218*** | Cristalino de Chihuahua>Azul | Mexico | 1964 | 28.85 | -107.75 |
| 130 | ***CHIH 38*** | Apachito | Mexico | 1800 | 27.80 | -107.48 |
| 131 | ***CHIS 104*** | Zapalote Grande | Mexico | 100 | 15.33 | -92.67 |
| 132 | ***CHIS 94*** | Comiteco | Mexico | 1800 | 16.25 | -92.13 |
| 133 | ***MEXI5/CHIH135*** | Palomero Toluqueño | Mexico | 2652 | 19.283 | -99.667 |
| 134 | ***MEXI 7*** | Cacahuacintle | Mexico | 2652 | 19.283 | -99.633 |
| 135 | ***MEXI 726*** | Chalqueño | Mexico | 2700 | 19.283 | -99.650 |
| 136 | ***MICH 362*** | Mushito | Mexico | 2340 | 19.667 | -102.338 |
| 137 | ***NAYA 24*** | Harinoso de Ocho | Mexico | 100 | 21.97 | -105.28 |
| 138 | ***OAXA 50*** | Zapalote Chico | Mexico | 100 | 16.58 | -94.60 |
| 139 | ***PI 217413*** | Zapalote Chico | Mexico | 2220 | 19.383 | -99.150 |
| 140 | ***PUEB 70*** | Cónico | Mexico | 2469 | 18.87 | -97.40 |
| 141 | ***PUEB 91*** | Arrocillo Amarillo | Mexico | 2060 | 19.750 | -97.550 |
| 142 | ***SINA 6*** | Chapalote | Mexico | 75 | 26.433 | -108.633 |
| 143 | ***SONO 24*** | Onaveño | Mexico | 1640 | 30.90 | -110.61 |
| 144 | ***TLAX 151*** | Conico | Mexico | 2463 | 19.30 | -97.77 |
| 145 | ***VERA 359*** | Arrocillo Amarillo | Mexico | 2200 | 19.77 | -97.23 |
| 146 | ***YUCA GP2*** | Nal-Tel>Harinoso | Mexico | 30 | 20.25 | -89.65 |
| 147 | ***CHIS 662*** | Zapalote Chico | Mexico | 100 | 16.217 | -93.889 |
| 148 | ***SINA 2*** | Chapalote | Mexico | 61 | 24.83 | -107.36 |
| 149 | ***OAXA 223*** | Bolita | Mexico | 200 | 15.750 | -96.517 |
| 150 | ***MEXI 3*** | Conico | Mexico | 2652 | 19.283 | -99.650 |
| 151 | ***VERA 39*** | Tuxpeño | Mexico | 106 | 20.47 | -97.08 |
| 152 | ***CHIS 63*** | Tepecintle | Mexico | 760 | 16.767 | -93.38 |
| 153 | ***GUER GP25*** | Vandeno | Mexico | 1000 | 17.52 | -101.28 |
| 154 | ***JALI 71*** | Elotes Occidentales | Mexico | 1555 | 20.17 | -103.03 |
| 155 | ***NAYA 337K*** | Jala | Mexico | 1080 | 21.09 | -104.43 |
| 156 | ***NAYA 39*** | Reventador | Mexico | 100 | 21.95 | -105.28 |
| 157 | ***OAXA GP1*** | Olotillo | Mexico | 120 | 16.33 | -98.03 |
| 158 | ***CHIH 160*** | Gordo>Cristalino de Chihuahua | Mexico | 2000 | 28.55 | -107.47 |
| 159 | ***ZACA 12*** | Cónico Norteño | Mexico | 1950 | 21.350 | -102.800 |
| 160 | ***JALI 102*** | Tabloncillo | Mexico | 1280 | 19.45 | -103.28 |
| 161 | ***JALI 78*** | Maíz Dulce | Mexico | 1890 | 21.18 | -102.85 |
| 162 | ***MEXI 212*** | Cacahuacintle | Mexico | 2800 | 19.07 | -99.45 |
| 163 | ***PANA 168*** | Salvadoreño>Clavillo | Panama | 213 | 8.22 | -81.70 |
| 164 | ***PAZM 13041*** | Avati' Moroti' Ti' | Paraguay | 230 | -23.000 | -56.000 |
| 165 | ***PARAGU CB6-62*** | Avati' Pichinga | Paraguay | 300 | -27.000 | -57.000 |
| 166 | ***PARAGU PG6-42*** | Avati' Moroti' | Paraguay | 400 | -26.1 | -55.933 |
| 167 | ***PAZM 6060*** | Avati | Paraguay | 240 | -26.267 | -56.117 |
| 168 | ***ANC. 212*** | Cuzco | Peru | 3100 | -9.33 | -77.23 |
| 169 | ***ANC 393*** | San Jeronimo>Huancavelicano | Peru | 2600 | -9.29 | -77.64 |
| 170 | ***APUC 140*** | Confite Puneño | Peru | 2500 | -13.62 | -72.88 |
| 171 | ***APUC 171*** | San Jeronimo | Peru | 2300 | -13.62 | -72.88 |
| 172 | ***PERU 674*** | Uchuquilla | Peru | 2025 | -15.83 | -70.03 |
| 173 | ***PERU 1303*** | Confite Morocho | Peru | 2744 | -12.77 | -75.03 |
| 174 | ***PERU 1283*** | Huancavelicano | Peru | 2698 | -12.77 | -75.03 |
| 175 | ***CUZ. 363*** | Cuzcri | Peru | 3200 | -13.46 | -72.16 |
| 176 | ***CUZ. 56*** | Cuzco Cristalino Amarillo | Peru | 3268 | -13.55 | -71.89 |
| 177 | ***ANC. 186*** | Ancashino | Peru | 2900 | -9.43 | -77.25 |
| 178 | ***LBQUE. 7*** | Mochero | Peru | 23 | -6.7 | -79.917 |
| 179 | ***HVCA. 69*** | Chulpi | Peru | 2800 | -12.4 | -74.9 |
| 180 | ***LIM. 47*** | Perla | Peru | 253 | -12.083 | -76.95 |
| 181 | ***CAJ. 24*** | Kkulli | Peru | 2300 | -7.167 | -78.517 |
| 182 | ***SVIN 5*** | Coastal Tropical Flint | St Vicent | 80 | 13.28 | -61.12 |
| 183 | ***SVIN GP2A*** | Coastal Tropical Flint | St Vicent | 100 | 13.28 | -61.24 |
| 184 | ***TRIN 6*** | Tusón | Trinidad and Tobago | 31 | 10.651 | -61.400 |
| 185 | ***URUG 697*** | Cuarenton >Colorado Cateto | Uruguay | 183 | -33.800 | -56.833 |
| 186 | ***URUG 8A*** | Cateto Sulino | Uruguay | 20 | -34.455 | -56.617 |
| 187 | ***URUGUA 1131A*** | Cateto Sulino | Uruguay | 108 | -34.167 | -57.5 |
| 188 | ***URUGUA 1187A*** | Dente Branco | Uruguay | 5 | -32.683 | -58.133 |
| 189 | ***URUGUA 637*** | Cateto Sulino | Uruguay | 80 | -34.34 | -57.73 |
| 190 | ***VEN 736*** | Sabanero | Venezuela | 2468 | 8.75 | -71.33 |
| 191 | ***VENEZU 631*** | Cariaco | Venezuela | 365 | 6.333 | -63.5 |
| 192 | ***VEN 650*** | Cuba Yellow Flint | Venezuela | 137 | 8.90 | -64.22 |
| 193 | ***VEN 405*** | Tusón | Venezuela | 425 | 7.58 | -71.97 |
| 194 | ***VEN 442*** | Tusón | Venezuela | 996 | 10.267 | -67.083 |
