# Supplementary material for: Genetic diversity and population structure of native maize populations in Latin America and the Caribbean
Source: PLoS One. 2017 Apr 12;12(4):e0173488. doi: 10.1371/journal.pone.0173488 (PMC5389613; doi:10.1371/journal.pone.0173488)
Supplement: S2 Table — a SSR location in the genome. b SSR repeat unit. c Allele size range in bp over the whole dataset. Loci§ in common with global diffusion of maize study [7]. (DOCX) [file pone.0173488.s008.docx]

**Table S2: List of the 28 SSR loci used to characterize the landraces in this study**.

| **Locus** | **Bin^a^** | **Motif^b^** | **Allele** |
| --- | --- | --- | --- |
|  |  |  | **range (bp)^c^** |
| ***phi109275*** | 1 | AGCT | 108-138 |
| ***umc2047*** | 1.09 | GACT | 112-143 |
| ***phi308707^§^*** | 1.1 | AGC | 111-135 |
| ***phi227562^§^*** | 1.12 | ACC | 292-343 |
| ***phi083^§^*** | 2.04 | AGCT | 114-163 |
| ***phi029^§^*** | 3.04 | AG/AGCG | 137-181 |
| ***umc1266*** | 3.06 | CAG | 117-149 |
| ***phi046^§^*** | 3.08 | ACGC | 58-74 |
| ***phi072^§^*** | 4.01 | AAAC | 128-164 |
| ***phi076*** | 4.11 | GAGCGG | 153-177 |
| ***phi109188*** | 5 | AAAG | 141-181 |
| ***umc1332*** | 5.04 | CTA | 107-146 |
| ***phi331888^§^*** | 5.04 | AAG | 124-138 |
| ***phi085^§^*** | 5.07 | AACGC | 229-265 |
| ***phi075*** | 6 | CT | 205-253 |
| ***phi031^§^*** | 6.04 | GTAC | 178-234 |
| ***phi299852*** | 6.08 | AGC | 95-150 |
| ***phi034*** | 7 | CCT | 110-149 |
| ***phi069^§^*** | 7.05 | GAC | 185-206 |
| ***phi115^§^*** | 8.03 | AT/ATAC | 290-310 |
| ***phi014^§^*** | 8.04 | GGC | 142-178 |
| ***phi108411^§^*** | 9.06 | AGCT | 111-137 |
| ***phi059^§^*** | 10.02 | ACC | 117-161 |
| ***phi063*** | 10.02 | TATC | 150-226 |
| ***phi062^§^*** | 10.04 | ACG | 155-179 |
| ***phi084^§^*** | 10.04 | GAA | 146-179 |
| ***umc1196*** | 10.07 | CACACG | 129-165 |
| ***phi102228^§^*** | 3.04-05 | AAGC | 119-131 |
