# Supplementary material for: Genetic diversity and population structure of native maize populations in Latin America and the Caribbean
Source: PLoS One. 2017 Apr 12;12(4):e0173488. doi: 10.1371/journal.pone.0173488 (PMC5389613; doi:10.1371/journal.pone.0173488)
Supplement: S3 Table — Classification of accessions according to 5 previous studies. ᵃResults of this study based on the structure analysis for K = 4, group 1 corresponds to maize accessions from Sierra Madre Occidental, group 2 to Southern Mexico, group 3 to Central Valleys, and group 4 to Chapalote Complex; ᵇResults of this study based on Neighbor Joining (NJ) cluster analysis; ᶜRacial Complexes, classification based on a combination of morphological, cytological and isozyme data [1]; ᵈClassification based on microsatellite data [8]; ᵉClassification based on a combination of morphological and isozyme data [9]; ᶠClassification based on cob morphological characteristics [10]; ᶢClassification based on morphological data [11]; ng: race not clustered to a specific group, np: race not included in the study. (DOCX) [file pone.0173488.s009.docx]

**Table S3: Mexican race names, accession, and altitude of the regions where the accessions were grown. Classification of accessions according to 5 previous studies.**

| **Race name** | **Accession** | **Altitude (m)** | **Aᵃ** | **Bᵇ** | **Cᶜ** | **Dᵈ** | **Eᵉ** | **Fᶠ** | **Gᶢ** |
| --- | --- | --- | --- | --- | --- | --- | --- | --- | --- |
| Apachito | CHIH 38 | 1800 | 1 | 1 | 1 | np | 3 | 3 | np |
| Arrocillo Amarillo | PUEB 91 | 2060 | 3 | 3 | 3 | 3 | 3 | 3 | 3 |
|  | VERA 359 | 2200 | 3 | 3 |  |  |  |  |  |
| Azul/Cristalino de Chihuahua | CHIH 133 | 2095 | 1 | 1 | 1 | np | 3 | 3 | np |
| Bolita | OAXA 223 | 200 | ng | 1 | 2 | 2 | 1 | 1 | 1 |
| Cacahuacintle | MEXI 7 | 2562 | 3 | 3 | 3 | 3 | 3 | 3 | 3 |
|  | MEXI 212 | 2800 | 3 | 3 | 3 |  |  |  |  |
| Chalqueño | MEXI 726 | 2700 | 3 | 3 | 3 | 3 | 3 | 3 | 3 |
| Chapalote | SINA 6 | 75 | 1 | 1 | 1 | 1 | 2 | 4 | 4 |
|  | SINA 2 | 61 | 4 | 1 | 1 |  |  |  |  |
| Comiteco | CHIS 94 | 1800 | 2 | 2 | 2 | 2 | 2 | 2 | 2 |
| Cónico | PUEB 70 | 2469 | 3 | 3 | 3 | 3 | 3 | 3 | 3 |
|  | TLAX 151 | 2463 | 3 | 3 | 3 |  |  |  |  |
|  | MEXI 3 | 2652 | 3 | 3 | 3 |  |  |  |  |
| Cónico Norteño | ZACA 12 | 1950 | 3 | 3 | 3 | 3 | 3 | 3 | 3 |
| Cristalino de Chihuahua/Apachito | CHIH 207 | 2510 | 1 | 1 | 1 | np | 3 | 3 | np |
| Cristalino de Chihuahua/Azul | CHIH 218 | 1964 | 1 | 1 | 1 | np | 3 | 3 | np |
| Elotes Occidentales | JALI 71 | 1555 | 4 | 1 | np | np | 1 | 1 | np |
| Gordo | CHIH 131 | 2095 | 1 | 1 | 1 | np | 3 | 3 | np |
| Gordo/Cristalino de Chihuahua | CHIH 160 | 2000 | 1 | 1 | 1 | np | 3 | 3 | np |
| Harinoso de Ocho | NAYA 24 | 100 | 1 | 2 | 1 | 1 | 1 | np | np |
| Jala | NAYA 337 | 1080 | 1 | 1 | 2 | 2 | 1 | 1 | 1 |
| Maíz Dulce | JALI 78 | 1890 | ng | 1 | 3 | 3 | 3 | 3 | 3 |
| Mushito | MICH 362 | 2340 | 3 | 3 | np | np | 2 | np | np |
| Nal-Tel | YUCA GP2 | 30 | 2 | 2 | 2 | 2 | 2 | 2 | 2 |
| Olotillo | OAXA GP1 | 120 | 1 | 2 | 2 | 2 | 2 | 2 | 2 |
| Onaveño | SONO 24 | 1640 | 1 | 1 | 1 | np | 1 | 1 | np |
| Palomero Toluqueño/Cristalino de Chihuahua | CHIH150 | 2140 | 1 | 1 | 1 | np | 3 | 3 | np |
| Palomero Toluqueño | MEXI 5 | 2652 | 3 | 3 | 3 | 3 | 3 | 3 | 3 |
| Reventador | NAYA 39 | 100 | 4 | 1 | 1 | 1 | 2 | 4 | 4 |
| Tabloncillo | JALI 102 | 1280 | 1 | 1 | 1 | 2 | 1 | 1 | 1 |
| Tepecintle | CHIS 63 | 760 | 2 | 2 | 2 | 2 | 2 | 2 | 2 |
| Tuxpeño | VERA 39 | 106 | 2 | 2 | 2 | 2 | 2 | 2 | 2 |
| Vandeño | GUER GP25 | 1000 | 2 | 2 | 2 | np | 2 | 2 | 2 |
| Zapalote Chico | OAXA 50 | 100 | 2 | 2 | 2 | 2 | 2 | 2 | 2 |
|  | PI 217413 | 100 | 2 | 2 | 2 |  |  |  |  |
|  | CHIS 662 | 100 | 2 | 2 | 2 |  |  |  |  |
| Zapalote Grande | CHIS 104 | 100 | 2 | 2 | 2 | 2 | 2 | 2 | 2 |
