# Supplementary material for: Genetic diversity and population structure of native maize populations in Latin America and the Caribbean
Source: PLoS One. 2017 Apr 12;12(4):e0173488. doi: 10.1371/journal.pone.0173488 (PMC5389613; doi:10.1371/journal.pone.0173488)
Supplement: S4 Table — Codes used and correspondences between clusters inferred with the most representative accessions with the study mentioned, and structure and sub-structure patterns in the present study, ng populations not grouped at structure or sub-structure level. (DOCX) [file pone.0173488.s010.docx]

**Table S4: Accessions in common with the study: Out of America: tracing the genetic footprints of the global diffusion of maize [7]**

| **ID** | **Accession** | **Accessions code in Mir et al. 2013** | **Cluster inferred with the most representative accessions by Mir et al. 2013** | **Structure analysis in this study** | **Sub structure analysis in this study** | **Name of subgroups in substructure analysis** |
| --- | --- | --- | --- | --- | --- | --- |
| 9 | ARZM 03 013 | Arg4 |  | Mexico and southern Andes | g1 | Northern Mexico |
| 125 | CHIH 131 | Mex6 |  | Mexico and southern Andes | g1 | Northern Mexico |
| 126 | CHIH 133 | Mex7 |  | Mexico and southern Andes | g1 | Northern Mexico |
| 127 | CHIH 150 | Mex8 |  | Mexico and southern Andes | g1 | Northern Mexico |
| 128 | CHIH 207 | Mex10 |  | Mexico and southern Andes | g1 | Northern Mexico |
| 129 | CHIH 218 | Mex11 | *Mexican highlands* | Mexico and southern Andes | g1 | Northern Mexico |
| 130 | CHIH 38 | Mex12 |  | Mexico and southern Andes | g1 | Northern Mexico |
| 142 | SINA 6 | Mex37 |  | Mexico and southern Andes | g1 | Northern Mexico |
| 143 | SONO 24 | Mex38 |  | Mexico and southern Andes | g1 | Northern Mexico |
| 148 | SINA 2 | Mex36 | *Mexican highlands* | Mexico and southern Andes | g1 | Northern Mexico |
| 154 | JALI 71 | Mex19 | *Mexican highlands* | Mexico and southern Andes | g1 | Northern Mexico |
| 155 | NAYA 337K | Mex28 |  | Mexico and southern Andes | g1 | Northern Mexico |
| 156 | NAYA 39 | Mex29 |  | Mexico and southern Andes | g1 | Northern Mexico |
| 158 | CHIH 160 | Mex9 | *Mexican highlands* | Mexico and southern Andes | g1 | Northern Mexico |
| 160 | JALI 102 | Mex18 |  | Mexico and southern Andes | g1 | Northern Mexico |
| 161 | JALI 78 | Mex20 |  | Mexico and southern Andes | g1 | Northern Mexico |
| 188 | URUGUA 1187A | Uru2 |  | Mexico and southern Andes | g1 | Northern Mexico |
| 133 | MEXI5/CHIH135 | Mex23 |  | Mexico and southern Andes | g2 | Central Mexico |
| 134 | MEXI 7 | Mex24 | *Mexican highlands* | Mexico and southern Andes | g2 | Central Mexico |
| 135 | MEXI 726 | Mex25 |  | Mexico and southern Andes | g2 | Central Mexico |
| 136 | MICH 362 | Mex26 |  | Mexico and southern Andes | g2 | Central Mexico |
| 137 | NAYA 24 | Mex27 |  | Mexico and southern Andes | g2 | Central Mexico |
| 140 | PUEB 70 | Mex34 |  | Mexico and southern Andes | g2 | Central Mexico |
| 141 | PUEB 91 | Mex35 |  | Mexico and southern Andes | g2 | Central Mexico |
| 144 | TLAX 151 | Mex39 |  | Mexico and southern Andes | g2 | Central Mexico |
| 145 | VERA 359 | Mex40 |  | Mexico and southern Andes | g2 | Central Mexico |
| 150 | MEXI 3 | Mex22 |  | Mexico and southern Andes | g2 | Central Mexico |
| 159 | ZACA 12 | Mex43 |  | Mexico and southern Andes | g2 | Central Mexico |
| 162 | MEXI 212 | Mex21 | *Mexican highlands* | Mexico and southern Andes | g2 | Central Mexico |
| 5 | PPS920 | Arg10 | *Northern US Flints* | Mexico and southern Andes | g3 | Southern Andes |
| 6 | ARGENT 24 | Arg2 |  | Mexico and southern Andes | g3 | Southern Andes |
| 43 | PPS1066 | Chi7 |  | Mexico and southern Andes | g3 | Southern Andes |
| 44 | PPS938 | Chi9 |  | Mexico and southern Andes | g3 | Southern Andes |
| 45 | PPS941 | Chi11 |  | Mexico and southern Andes | g3 | Southern Andes |
| 46 | PPS949 | Chi12 |  | Mexico and southern Andes | g3 | Southern Andes |
| 47 | PPS961 | Chi13 | *Northern US Flints* | Mexico and southern Andes | g3 | Southern Andes |
| 48 | PPS939 | Chi10 |  | Mexico and southern Andes | g3 | Southern Andes |
| 49 | CHZM 08 060 | Chi5 |  | Mexico and southern Andes | g3 | Southern Andes |
| 50 | PPS55 | Chi8 | *Northern US Flints* | Mexico and southern Andes | g3 | Southern Andes |
| 51 | CHZM 09 030 | Chi4 | *Northern US Flints* | Mexico and southern Andes | g3 | Southern Andes |
| 53 | CHZM 08 049 | Chi2 | *Northern US Flints* | Mexico and southern Andes | g3 | Southern Andes |
| 86 | ECUA 500 | Ecu1 |  | Mexico and southern Andes | g3 | Southern Andes |
| 87 | ECUA 696 | Ecu17 |  | Mexico and southern Andes | g3 | Southern Andes |
| 170 | APUC 140 | Per13 |  | Mexico and southern Andes | g3 | Southern Andes |
| 171 | APUC 171 | Per14 |  | Mexico and southern Andes | g3 | Southern Andes |
| 187 | URUGUA 1131A | Uru3 |  | Mexico and southern Andes | g3 | Southern Andes |
| 18 | BOLI 711 | Bol16 |  | Mesoamerica lowland | g4 | Tropical lowland |
| 39 | BRVI 117 | Vir2 |  | Mesoamerica lowland | g4 | Tropical lowland |
| 59 | ANTI 392 | Col22 |  | Mesoamerica lowland | g4 | Tropical lowland |
| 74 | CRIC 141 | Cos1 |  | Mesoamerica lowland | g4 | Tropical lowland |
| 75 | CRIC 166 | Cos2 |  | Mesoamerica lowland | g4 | Tropical lowland |
| 76 | CUBA 44 | Cub3 |  | Mesoamerica lowland | g4 | Tropical lowland |
| 77 | CUBA 54 | Cub4 |  | Mesoamerica lowland | g4 | Tropical lowland |
| 78 | CUBA 63 | Cub5 |  | Mesoamerica lowland | g4 | Tropical lowland |
| 79 | CUBA 85 | Cub1 |  | Mesoamerica lowland | g4 | Tropical lowland |
| 85 | ECUA 617 | Ecu16 |  | Mesoamerica lowland | g4 | Tropical lowland |
| 91 | ECUA 881 | Ecu18 |  | Mesoamerica lowland | g4 | Tropical lowland |
| 102 | GUAD 6 | Gdp6 |  | Mesoamerica lowland | g4 | Tropical lowland |
| 108 | GUAT 162 | Gua11 |  | Mesoamerica lowland | g4 | Tropical lowland |
| 186 | URUG 8A | Uru6 |  | Mesoamerica lowland | g4 | Tropical lowland |
| 192 | VEN 650 | Ven5 |  | Mesoamerica lowland | g4 | Tropical lowland |
| 193 | VEN 405 | Ven3 |  | Mesoamerica lowland | g4 | Tropical lowland |
| 194 | VEN 442 | Ven4 |  | Mesoamerica lowland | g4 | Tropical lowland |
| 38 | BRVI 104 | Vir1 |  | Mesoamerica lowland | g5 | Mex/Gua/Vir/Ven |
| 109 | GUAT 36 | Gua12 |  | Mesoamerica lowland | g5 | Mex/Gua/Vir/Ven |
| 110 | GUAT 45 | Gua13 |  | Mesoamerica lowland | g5 | Mex/Gua/Vir/Ven |
| 111 | GUAT 527 | Gua14 |  | Mesoamerica lowland | g5 | Mex/Gua/Vir/Ven |
| 112 | GUAT 529 | Gua15 |  | Mesoamerica lowland | g5 | Mex/Gua/Vir/Ven |
| 113 | GUAT 606 | Gua16 |  | Mesoamerica lowland | g5 | Mex/Gua/Vir/Ven |
| 114 | GUAT 820 | Gua17 |  | Mesoamerica lowland | g5 | Mex/Gua/Vir/Ven |
| 120 | GUATEM 155 | Gua7 |  | Mesoamerica lowland | g5 | Mex/Gua/Vir/Ven |
| 131 | CHIS 104 | Mex13 |  | Mesoamerica lowland | g5 | Mex/Gua/Vir/Ven |
| 132 | CHIS 94 | Mex16 |  | Mesoamerica lowland | g5 | Mex/Gua/Vir/Ven |
| 138 | OAXA 50 | Mex31 | *Mexican highlands* | Mesoamerica lowland | g5 | Mex/Gua/Vir/Ven |
| 139 | PI 217413 | Mex33 |  | Mesoamerica lowland | g5 | Mex/Gua/Vir/Ven |
| 146 | YUCA GP2 | Mex42 |  | Mesoamerica lowland | g5 | Mex/Gua/Vir/Ven |
| 147 | CHIS 662 | Mex15 |  | Mesoamerica lowland | g5 | Mex/Gua/Vir/Ven |
| 151 | VERA 39 | Mex41 |  | Mesoamerica lowland | g5 | Mex/Gua/Vir/Ven |
| 152 | CHIS 63 | Mex14 |  | Mesoamerica lowland | g5 | Mex/Gua/Vir/Ven |
| 153 | GUER GP25 | Mex17 |  | Mesoamerica lowland | g5 | Mex/Gua/Vir/Ven |
| 190 | VEN 736 | Ven6 |  | Mesoamerica lowland | g5 | Mex/Gua/Vir/Ven |
| 40 | BRVI 139 | Vir3 | *Tropical lowlands* | Mesoamerica lowland | g6 | Lesser Antilles |
| 41 | BRVI 142 | Vir4 | *Tropical lowlands* | Mesoamerica lowland | g6 | Lesser Antilles |
| 124 | MART 4 | Mrt1 | *Tropical lowlands* | Mesoamerica lowland | g6 | Lesser Antilles |
| 182 | SVIN 5 | Svt1 |  | Mesoamerica lowland | g6 | Lesser Antilles |
| 1 | ANTI GP2 | Ant3 | *Tropical lowlands* | Mesoamerica lowland | g7 | Greater and Lesser Antilles |
| 36 | 26373 | Bra3 |  | Mesoamerica lowland | g7 | Greater and Lesser Antilles |
| 81 | RDOM 270 | Dom1 |  | Mesoamerica lowland | g7 | Greater and Lesser Antilles |
| 82 | RDOM GP1 | Dom2 |  | Mesoamerica lowland | g7 | Greater and Lesser Antilles |
| 100 | GFO053 | Gdp4 | *Tropical lowlands* | Mesoamerica lowland | g7 | Greater and Lesser Antilles |
| 101 | GFO059 | Gdp5 |  | Mesoamerica lowland | g7 | Greater and Lesser Antilles |
| 103 | STB073 | Gdp9 |  | Mesoamerica lowland | g7 | Greater and Lesser Antilles |
| 104 | EGT014 | Gdp3 |  | Mesoamerica lowland | g7 | Greater and Lesser Antilles |
| 105 | MGA201 | Gdp7 |  | Mesoamerica lowland | g7 | Greater and Lesser Antilles |
| 106 | DES106 | Gdp2 |  | Mesoamerica lowland | g7 | Greater and Lesser Antilles |
| 184 | TRIN 6 | Tri1 |  | Mesoamerica lowland | g7 | Greater and Lesser Antilles |
| 11 | BARB 5 | Bar1 |  | Mesoamerica lowland | ng |  |
| 12 | BARB GP2 | Bar2 |  | Mesoamerica lowland | ng |  |
| 13 | BARB GP1 | Bar3 |  | Mesoamerica lowland | ng |  |
| 80 | CUBA 12 | Cub2 |  | Mesoamerica lowland | ng |  |
| 99 | DES103 | Gdp1 | *Tropical lowlands* | Mesoamerica lowland | ng |  |
| 107 | MGA235 | Gdp8 |  | Mesoamerica lowland | ng |  |
| 163 | PANA 168 | Pan1 |  | Mesoamerica lowland | ng |  |
| 183 | SVIN GP2A | Svt2 | *Tropical lowlands* | Mesoamerica lowland | ng |  |
| 54 | NARINO 369 | Col4 |  | South America- Andean Region | g10 | Colombian 1 |
| 56 | TOLIMA 389 | Col15 | *Northern South-America* | South America- Andean Region | g10 | Colombian 1 |
| 57 | MAGDAL 443 | Col19 |  | South America- Andean Region | g10 | Colombian 1 |
| 60 | BOYACA 462 | Col16 | *Northern South-America* | South America- Andean Region | g10 | Colombian 1 |
| 63 | CAQUET 321 | Col11 |  | South America- Andean Region | g10 | Colombian 1 |
| 64 | CUNDIN 428 | Col14 |  | South America- Andean Region | g10 | Colombian 1 |
| 66 | COLOMB 613 | Col10 | *Northern South-America* | South America- Andean Region | g10 | Colombian 1 |
| 71 | CORDOB 342 | Col7 | *Northern South-America* | South America- Andean Region | g10 | Colombian 1 |
| 73 | SANTAS 340 | Col1 | *Northern South-America* | South America- Andean Region | g10 | Colombian 1 |
| 32 | BRAZIL 1546 | Bra7 | *Middle South-America* | South America- Andean Region | g11 | Colombian2 |
| 65 | CUNDIN 465 | Col20 |  | South America- Andean Region | g11 | Colombian2 |
| 67 | SANTAN 332 | Col13 |  | South America- Andean Region | g11 | Colombian2 |
| 72 | MAGDAL 390 | Col12 |  | South America- Andean Region | g11 | Colombian2 |
| 189 | URUGUA 637 | Uru4 |  | South America- Andean Region | g11 | Colombian2 |
| 3 | ARGE 564 | Arg8 |  | South America- Andean Region | g12 | Highland Andes |
| 10 | ARGENT TUC16 | Arg3 |  | South America- Andean Region | g12 | Highland Andes |
| 14 | BOLI 905 | Bol17 | *Andes* | South America- Andean Region | g12 | Highland Andes |
| 15 | BOLI 929 | Bol18 | *Andes* | South America- Andean Region | g12 | Highland Andes |
| 84 | ECUA 476 | Ecu14 | *Andes* | South America- Andean Region | g12 | Highland Andes |
| 90 | ECUA 418 | Ecu13 | *Andes* | South America- Andean Region | g12 | Highland Andes |
| 169 | ANC 393 | Per12 | *Andes* | South America- Andean Region | g12 | Highland Andes |
| 173 | PERU 1303 | Per16 | *Andes* | South America- Andean Region | g12 | Highland Andes |
| 83 | ECUADO 398 | Ecu7 | *Andes* | South America- Andean Region | g13 | Ecuadorian Highland |
| 88 | ECUADO 486 | Ecu9 | *Andes* | South America- Andean Region | g13 | Ecuadorian Highland |
| 93 | ECUADO 746 | Ecu5 |  | South America- Andean Region | g13 | Ecuadorian Highland |
| 95 | ECUADO 424 | Ecu4 |  | South America- Andean Region | g13 | Ecuadorian Highland |
| 96 | ECUADO 418 | Ecu8 | *Andes* | South America- Andean Region | g13 | Ecuadorian Highland |
| 97 | ECUADO X14237 | Ecu6 | *Andes* | South America- Andean Region | g13 | Ecuadorian Highland |
| 98 | ECUADO 459 | Ecu3 | *Andes* | South America- Andean Region | g13 | Ecuadorian Highland |
| 168 | ANC. 212 | Per7 | *Andes* | South America- Andean Region | g13 | Ecuadorian Highland |
| 8 | ARGENT TUC43 | Arg1 |  | South America- Andean Region | g14 | Central Highland Andean |
| 29 | BOZM 677 | Bol5 |  | South America- Andean Region | g14 | Central Highland Andean |
| 89 | ECUADO 704 | Ecu12 | *Andes* | South America- Andean Region | g14 | Central Highland Andean |
| 175 | CUZ. 363 | Per5 | *Andes* | South America- Andean Region | g14 | Central Highland Andean |
| 178 | LBQUE. 7 | Per11 | *Andes* | South America- Andean Region | g14 | Central Highland Andean |
| 55 | NARINO 392 | Col9 |  | South America- Andean Region | g15 | Northern tropical lowland |
| 62 | TOLIMA 390 | Col3 |  | South America- Andean Region | g15 | Northern tropical lowland |
| 69 | NARINO 534 | Col5 |  | South America- Andean Region | g15 | Northern tropical lowland |
| 121 | GUATEM 548 | Gua5 |  | South America- Andean Region | g15 | Northern tropical lowland |
| 123 | GUATEM 93 | Gua10 |  | South America- Andean Region | g15 | Northern tropical lowland |
| 191 | VENEZU 631 | Ven2 |  | South America- Andean Region | g15 | Northern tropical lowland |
| 20 | BOLIVI 1037 | Bol8 | *Middle South-America* | South America- Andean Region | g16 | Moroti's |
| 37 | BRAZIL 60 | Bra4 | *Middle South-America* | South America- Andean Region | g16 | Moroti's |
| 164 | PAZM 13041 | Par3 |  | South America- Andean Region | g16 | Moroti's |
| 165 | PARAGU CB6-62 | Par2 | *Middle South-America* | South America- Andean Region | g16 | Moroti's |
| 166 | PARAGU PG6-42 | Par4 | *Middle South-America* | South America- Andean Region | g16 | Moroti's |
| 167 | PAZM 6060 | Par6 | *Middle South-America* | South America- Andean Region | g16 | Moroti's |
| 21 | BOLIVI 928 | Bol9 | *Andes* | South America- Andean Region | g8 | Bolivian highlands |
| 23 | BOZM 1791 | Bol12 |  | South America- Andean Region | g8 | Bolivian highlands |
| 24 | BOZM 988 | Bol3 |  | South America- Andean Region | g8 | Bolivian highlands |
| 28 | BOZM 694 | Bol6 |  | South America- Andean Region | g8 | Bolivian highlands |
| 30 | BOZM 96 | Bol4 | *Andes* | South America- Andean Region | g8 | Bolivian highlands |
| 52 | CHZM 01 062 | Chi3 | *Andes* | South America- Andean Region | g8 | Bolivian highlands |
| 176 | CUZ. 56 | Per6 | *Andes* | South America- Andean Region | g8 | Bolivian highlands |
| 179 | HVCA. 69 | Per8 | *Andes* | South America- Andean Region | g8 | Bolivian highlands |
| 181 | CAJ. 24 | Per4 | *Andes* | South America- Andean Region | g8 | Bolivian highlands |
| 17 | BOZM 1609 | Bol10 | *Middle South-America* | South America- Andean Region | g9 | Bolivian lowland |
| 25 | BOLIVI 351 | Bol14 | *Middle South-America* | South America- Andean Region | g9 | Bolivian lowland |
| 27 | BOLIVI 90 | Bol15 | *Middle South-America* | South America- Andean Region | g9 | Bolivian lowland |
| 42 | CHZM 13 080 | Chi1 |  | South America- Andean Region | g9 | Bolivian lowland |
| 92 | ECUADO 573 | Ecu11 |  | South America- Andean Region | g9 | Bolivian lowland |
| 19 | BOLIVI 957 | Bol13 |  | South America- Andean Region | ng |  |
| 22 | BOLIVI 968 | Bol11 |  | South America- Andean Region | ng |  |
| 26 | BOZM 1681 | Bol7 |  | South America- Andean Region | ng |  |
| 177 | ANC. 186 | Per9 | *Andes* | South America- Andean Region | ng |  |
| 2 | ARGE 486 | Arg7 |  | ng |  |  |
| 4 | ARGE GP8 | Arg9 |  | ng |  |  |
| 7 | ARGENT 306 | Arg5 |  | ng |  |  |
| 16 | BOZMO 214 | Bol19 |  | ng |  |  |
| 31 | BRAZIL 2441 | Bra2 |  | ng |  |  |
| 33 | BRAZIL 2305 | Bra1 |  | ng |  |  |
| 34 | BRAZIL 953 | Bra5 |  | ng |  |  |
| 35 | BRAZIL PE012 | Bra6 |  | ng |  |  |
| 58 | CAUCA 384 | Col21 | *Northern South-America* | ng |  |  |
| 61 | CUNDIN 327 | Col18 | *Northern South-America* | ng |  |  |
| 68 | CUNDIN 480 | Col17 | *Northern South-America* | ng |  |  |
| 70 | SANTAN 317 | Col2 | *Northern South-America* | ng |  |  |
| 94 | ECUADO 979 | Ecu10 |  | ng |  |  |
| 115 | GUATEM 685 | Gua2 |  | ng |  |  |
| 116 | GUATEM SH22 | Gua8 |  | ng |  |  |
| 117 | GUATEM 213 | Gua9 |  | ng |  |  |
| 118 | GUATEM 390 | Gua4 |  | ng |  |  |
| 119 | GUATEM 404 | Gua3 | *Mexican highlands* | ng |  |  |
| 122 | GUATEM 10 | Gua6 |  | ng |  |  |
| 149 | OAXA 223 | Mex3 | *Mexican highlands* | ng |  |  |
| 157 | OAXA GP1 | Mex32 |  | ng |  |  |
| 172 | PERU 674 | Per17 |  | ng |  |  |
| 174 | PERU 1283 | Per15 | *Andes* | ng |  |  |
| 180 | LIM. 47 | Per10 |  | ng |  |  |
| 185 | URUG 697 | Uru5 |  | ng |  |  |
